# Southern African HIV Clinicians Society guidelines for antiretroviral therapy in adults: 2020 update

**DOI:** 10.4102/sajhivmed.v21i1.1115

**Published:** 2020-09-16

**Authors:** Jeremy Nel, Sipho Dlamini, Graeme Meintjes, Rosie Burton, John M. Black, Natasha E.C.G. Davies, Eric Hefer, Gary Maartens, Phetho M. Mangena, Moeketsi T. Mathe, Mahomed-Yunus Moosa, Muhangwi B. Mulaudzi, Michelle Moorhouse, Jennifer Nash, Thandeka C. Nkonyane, Wolfgang Preiser, Mohammed S. Rassool, David Stead, Helen van der Plas, Cloete van Vuuren, Willem D.F. Venter, Joana F. Woods

**Affiliations:** 1 Helen Joseph Hospital, Department of Medicine, University of the Witwatersrand, Johannesburg, South Africa; 2 Department of Infectious Diseases, Faculty of Medicine, University of Cape Town, Cape Town, South Africa; 3 Department of Medicine, Division of Infectious Diseases and HIV Medicine, Groote Schuur Hospital, University of Cape Town, Cape Town, South Africa; 4 Southern African Medical Unit, Médecins Sans Frontières (MSF), Cape Town, South Africa; 5 Department of Medicine, Division of Infectious Diseases, Livingstone Tertiary Hospital, Port Elizabeth, South Africa; 6 Anova Health Institute, Johannesburg ,South Africa; 7 Private Practice Medical Adviser, Johannesburg, South Africa; 8 Department of Medicine, Division of Clinical Pharmacology, University of Cape Town, Cape Town, South Africa; 9 Department of Internal Medicine, School of Medicine, Pietersburg Hospital, Polokwane, South Africa; 10 Department of Medicine, School of Medicine, University of Limpopo, Turfloop, South Africa; 11 Private Practice, Vereeniging, South Africa; 12 Department of Infectious Diseases, Division of Internal Medicine, University of KwaZulu-Natal, Durban, South Africa; 13 Private Practice (Phomolong Medical Centre), Rustenburg, South Africa; 14 Reproductive Health and HIV Institute, Faculty of Health Sciences, University of the Witwatersrand, Johannesburg, South Africa; 15 Specialist Family Physician, Amathole District Clinical Specialist Team, East London, South Africa; 16 Department of Infectious Diseases, Faculty of Medicine, Sefako Makgatho Health Sciences University, Pretoria, South Africa; 17 Department of Medicine, Dr George Mokhari Hospital, Pretoria, South Africa; 18 Department of Medical Virology, National Health Laboratory Service, Tygerberg, South Africa; 19 Department of Pathology, Faculty of Medicine and Health, Stellenbosch University, Cape Town, South Africa; 20 Clinical HIV Research Unit, Wits Health Consortium, Johannesburg, South Africa; 21 Department of Medicine, Faculty of Infectious Diseases, Frere and Cecilia Makiwane Hospitals, East London, South Africa; 22 Department of Medicine, Faculty of Health Sciences, Walter Sisulu University, Mthatha, South Africa; 23 Department of Internal Medicine, Military Hospital, Bloemfontein, South Africa; 24 Department of Internal Medicine, Faculty of Health Sciences, University of the Free State, Bloemfontein, South Africa; 25 Ezintsha, Faculty of Health Sciences, University of the Witwatersrand, Johannesburg, South Africa

## Table of contents

**What is new in the 2020 guidelines update?**

1. Preamble
2. Nucleoside or nucleotide reverse transcriptase inhibitor class of antiretroviral drugs
3. Integrase strand transfer inhibitor class of antiretroviral drugs
4. Non-nucleoside reverse transcriptase inhibitor class of antiretroviral drugs
5. Protease inhibitor class of antiretroviral drugs
6. Initiation and timing of antiretroviral therapy
7. Baseline investigations
8. Viral load
9. Cluster of differentiation 4 cell (CD4^+^) count
10. Resistance and genotyping
11. Initial antiretroviral therapy regimens for the previously untreated patient
12. Management of patients currently receiving first-line therapy
13. Management of patients starting or currently receiving second-line therapy
14. Third-line antiretroviral therapy
15. Laboratory monitoring of the efficacy and safety of antiretroviral therapy
16. Patients who return after stopping antiretroviral therapy
17. Drug–drug interactions
18. Tuberculosis
19. Pregnancy and breastfeeding
20. Liver disease
21. Renal disease
22. Psychiatric disease
23. Malaria
24. Antiretroviral drug-induced liver injury
25. Dyslipidaemia
26. Immune reconstitution inflammatory syndrome
27. Opportunistic infection prophylaxis
28. Adherence
29. Acknowledgments
30. Abbreviations
31. References

## What is new in the 2020 guidelines update?

### Key updates

➢ A recommendation for dolutegravir (DTG)-based therapies as the preferred first-line antiretroviral therapy (ART) option (section 11).
➢ Updated guidelines for second- and third-line ART regimens (section 13).
➢ New recommendations on the management of patients on DTG-based therapies who have an elevated viral load (section 12).
➢ A lowering of the threshold for virological failure from 1000 copies/mL to 50 copies/mL (section 8).
➢ A recommendation against routine cluster of differentiation 4 (CD4^+^) monitoring in patients who are clinically well once the CD4^+^ count is > 200 cells/µL (section 9).
➢ Updated recommendations for isoniazid preventive therapy (IPT) in human immunodeficiency virus (HIV)-positive patients (section 27).
➢ A recommendation for the use of low-dose prednisone as prophylaxis for paradoxical tuberculosis (TB) immune reconstitution inflammatory syndrome (IRIS) in TB/HIV co-infected patients commencing ART within 1 month of TB therapy (section 26).

## 1. Preamble

### Key principles

Although many antiretroviral therapy (ART) guidelines are available internationally, the current guidelines have been written to address issues relevant to southern Africa. A major spur for the current guidelines is the introduction of dolutegravir (DTG) into first- and second-line ART regimens. Dolutegravir-based ART regimens hold much promise, although the transition inevitably challenges existing paradigms and generates additional complexities. These guidelines aim to address many of these and to update the text in general to reflect the latest evidence.

As with previous iterations, these guidelines take affordability into account, as countries in the region vary according to their low- and middle-income status. Hence, only the treatment and diagnostic options that are available in southern Africa are included. In addition, these guidelines recognise the need to bridge the gap in treatment recommendations between public and private sector programmes, considering that many patients transition between the two sectors for treatment. The format of this iteration of the guidelines has been modified to highlight each section’s *key points* and *common pitfalls*. It will also be released over time as multiple stand-alone modules, designed with an emphasis on readability, ease of access and user-friendliness.

### Goals of antiretroviral therapy

The goals of ART are to:

- provide maximal and durable suppression of viral load (VL)
- restore and/or preserve immune function
- reduce human immunodeficiency virus (HIV)-related infectious and non-infectious morbidity
- prolong life expectancy and improve quality of life
- prevent onward transmission of HIV
- minimise adverse effects of the treatment

These goals are achieved by suppressing viral replication completely for as long as possible, using well-tolerated and sustainable treatment undertaken with good adherence. With prolonged viral suppression, the cluster of differentiation 4 (CD4^+^) lymphocyte count usually increases, which is accompanied by a restoration of pathogen-specific immune function. For most patients, this results in a dramatic reduction in the risk of HIV-associated morbidity and mortality. In patients who start to receive ART with preserved CD4^+^ counts, ART is able to prevent the decline in CD4^+^ count observed in untreated patients and prevent clinical complications of HIV infection. It is still unclear whether immune function ever returns to full normality, although long-term cohorts have shown that patients who adhere well to ART have a near-normal life expectancy.^1^ Patient adherence to the ART regimen remains a key focus and challenge.

### Stopping antiretroviral therapy

Antiretroviral therapy should not be stopped unless there is an extremely compelling reason to do so. In most cases where drug toxicities develop, switching the culprit drug(s) should be attempted instead. If non-nucleoside reverse transcriptase inhibitor (NNRTI)-based therapy is stopped, then we generally do not recommend ‘covering the tail’ with an additional 5–7 days of nucleoside reverse transcriptase inhibitors (NRTIs). There is little evidence for this in patients on long-term ART, and the intracellular half-life of drugs, such as tenofovir disoproxil fumarate (TDF), in any case approximates that of the NNRTIs.^2^ It is important to ensure that the VL is suppressed before substituting a single drug for toxicity; otherwise, resistance may develop to the new drug, consequently compromising future regimens. However, single-drug substitutions can be done in the first few months of ART without measuring the VL, as the VL may take up to 6 months to become suppressed.

We strongly advise against lamivudine (3TC) monotherapy ‘holding regimens’ in patients who have virological failure. Such regimens can be associated with a rapid fall in CD4^+^ count. When prescribing ART, the objective should always be to provide a regimen that could achieve virological suppression.

## 2. Nucleoside or nucleotide reverse transcriptase inhibitor class of antiretroviral drugs

### Key points

➢ The recommended nucleoside or nucleotide reverse transcriptase inhibitor (NRTI) drugs for first-line therapy are tenofovir disoproxil fumarate (TDF) and either 3TC or emtricitabine (FTC).
➢ Patients with a creatinine clearance rate (CrCl) < 50 mL/min should generally be started on abacavir (ABC) instead of TDF for first-line therapy.
➢ Zidovudine (AZT) should only be used in special circumstances as a first-line drug.
➢ Tenofovir disoproxil fumarate can cause renal failure or a renal-tubular wasting syndrome. Creatinine monitoring at regular intervals is recommended.
➢ Abacavir can cause a fatal hypersensitivity reaction in patients with HLA-B*5701. If feasible, this allele should be excluded prior to starting ABC, although it is very rare in people of African descent.
➢ Zidovudine can cause anaemia and neutropenia, and regular monitoring of haemoglobin (Hb) and neutrophil counts is recommended for the first 6 months.

### Available nucleoside or nucleotide reverse transcriptase inhibitors

Nucleoside reverse transcriptase inhibitors and nucleotide reverse transcriptase inhibitors (NtRTIs) work by acting as nucleotide base analogues. Following incorporation into the deoxyribonucleic acid (DNA) chain by HIV’s reverse transcriptase enzyme, they block further chain elongation. A summary of NRTIs is provided in Table 1, and the appropriate baseline investigations and required monitoring are presented in Table 2. Nucleoside reverse transcriptase inhibitors may be available as single tablets or in fixed-dose combination (FDC). The latter is recommended where possible to decrease the overall pill burden. Many NRTIs require dose adjustment in patients with renal failure (see section 21).

**TABLE 1:** Dosage and common adverse drug reactions of nucleoside or nucleotide reverse transcriptase inhibitors available in southern Africa (adult dosing).

Generic name	Class of drug	Recommended dosage	Common or severe ADR‡
Tenofovir disoproxil fumarate (TDF)	NtRTI	300 mg daily	**Renal failure**, tubular wasting syndrome, reduced bone mineral density, nausea
Lamivudine (3TC)	NRTI	150 mg 12 hourly or 300 mg daily	**Anaemia (pure red cell aplasia)** (rare)
Emtricitabine (FTC)†	NRTI	200 mg daily	**Anaemia (pure red cell aplasia)** (rare), palmar hyperpigmentation
Abacavir (ABC)	NRTI	300 mg 12 hourly or 600 mg daily	Hypersensitivity reaction
Zidovudine (AZT)	NRTI	300 mg 12 hourly	**Anaemia, neutropenia**, gastrointestinal upset, headache, myopathy, hyperlactataemia/steatohepatitis (medium potential), lipoatrophy

ADR, adverse drug reaction; NtRTI, nucleotide reverse transcriptase inhibitor; NRTI, nucleoside reverse transcriptase inhibitor.

† , FTC is not available as a single drug in South Africa, only co-formulated.

‡ , Life-threatening reactions are indicated in **bold**.

**TABLE 2:** Baseline investigations and monitoring required for nucleoside or nucleotide reverse transcriptase inhibitors.

Generic name	Monitoring required	Comment
Tenofovir disoproxil fumarate (TDF)	Creatinine before initiation, then at 3 months, 6 months and then 6 monthly thereafter	Avoid if eGFR < 50 mL/min In high-risk patients (particularly those with co-existent hypertension or diabetes), creatinine should also be checked at 1 and 2 months.
Lamivudine (3TC)	None routinely required	
Emtricitabine (FTC)	None routinely required	
Abacavir (ABC)	HLA-B*5701 before initiation, if testing is affordable and available	Allele very rare in people of African descent
Zidovudine (AZT)	Hb and neutrophil count before initiation, then at months 1, 2, 3 and 6	Avoid if Hb < 8 g/dL If neutrophil count is 1–1.5 × 10^9^, then repeat in 4 weeks. If neutrophil count is 0.75–0.99 × 10^9^, then repeat in 2 weeks or consider switching from AZT. If neutrophil count is < 0.75, then switch from AZT.

eGFR, estimated glomerular filtration rate; Hb, haemoglobin.

#### Lamivudine and emtricitabine

Lamivudine and FTC are well-tolerated drugs recommended as part of a first-line regimen. Although there are minor differences between them, 3TC and FTC are considered functionally interchangeable. Their use may be continued in the presence of ‘high-level resistance’ caused by the M184V mutation because this mutation impairs the replication ability of HIV, causing a ~0.5 log decrease in VL. Therefore, the drugs are often used in second- and third-line therapies (see the management of patients on second-line ART in section 13). Lamivudine and FTC are active against hepatitis B, but when used in the absence of a second drug active against hepatitis B, such as TDF, then resistance rates of approximately 50% at 1 year, and 90% at 5 years, are seen^3^. See section 20.

#### Tenofovir disoproxil fumarate

Tenofovir disoproxil fumarate is the preferred drug in this class for use with 3TC or FTC in first-line therapy because it aligns with public sector programmes, is widely available as an FDC and is generally well tolerated. Tenofovir disoproxil fumarate also offers durable therapy against hepatitis B virus (HBV). Hepatitis B virus resistance against TDF is extremely rare. In a minority of patients, TDF may cause a tubular wasting syndrome (including wasting of phosphate and potassium).^4^ If patients receiving TDF develop muscle weakness or other muscle symptoms, then potassium and phosphate levels must be assessed. Tenofovir disoproxil fumarate can also cause acute and chronic renal failure, but this is uncommon.^5^

Tenofovir disoproxil fumarate should be switched to ABC or an alternative NRTI immediately in patients with acute renal failure, as it may exacerbate injury even if it is not the primary cause. Consider recommencing TDF with careful monitoring when creatinine level is normal if an alternative cause of renal failure is established.

We recommend estimating the CrCl before commencing TDF; the drug should not be used if the estimated glomerular filtration rate (eGFR) or CrCl is < 50 mL/min. For monitoring whilst on TDF, see Table 2. Where TDF is avoided because CrCl is < 50 mL/min at baseline, it may be possible to switch to TDF at a later point if renal function improves. This is often the case where patients had diarrhoea or other opportunistic infections (OIs) at the time of ART initiation.

◦ **Common pitfall: Permanently discontinuing TDF in patients with transiently decreased CrCl.** Most cases of acute kidney injury (AKI) are not because of TDF, and if another cause of AKI is identified (e.g. severe diarrhoea or pneumonia), then TDF can be re-introduced with monitoring once renal function improves.

The long-term use of TDF together with other nephrotoxic agents (e.g. aminoglycosides or non-steroidal anti-inflammatory agents) should be avoided. Tenofovir disoproxil fumarate also causes a decrease in bone mineral density; however, this is generally mild and non-progressive, and most studies have not found an increase in fracture risk.

#### Abacavir

Abacavir can be used in patients with a CrCl < 50 mL/min at baseline, rather than TDF. Abacavir does not require dose adjustment in patients with renal failure and is especially useful in patients with chronic renal failure, where TDF is nephrotoxic and AZT could aggravate anaemia in patients with renal failure. A meta-analysis showed that virological suppression is equivalent with ABC- and TDF-containing first-line regimens regardless of baseline VL.^6^

◦ **Common pitfall: Avoiding ABC at high HIV VLs.** This is unnecessary as viral suppression rates are equivalent in meta-analyses.

Abacavir has been associated with an increased risk of myocardial infarction in some but not all cohort studies; however, the association was not confirmed in a meta-analysis of randomised controlled trials (RCTs).^6,7,8^ Nevertheless, caution is recommended when considering ABC for patients who are at significant risk of or have established ischaemic heart disease. Abacavir hypersensitivity is a systemic reaction occurring within the first 8 weeks of therapy in approximately 3% of cases. Fatalities may occur on rechallenge. *Abacavir must be discontinued and never re-introduced if hypersensitivity is suspected*. The manifestations of hypersensitivity include fever, rash, fatigue and abdominal or respiratory symptoms. If there is any doubt concerning the diagnosis (e.g. if the patient has a cough with fever), then the patient should be admitted for observation of the next dose; symptoms progress if hypersensitivity is present. The hypersensitivity reaction has been shown to occur on a genetic basis, with a very strong association with the HLA-B*5701 allele. This allele is very uncommon in people of African descent; thus, ABC hypersensitivity is less frequent. If testing is affordable and available, then the presence of HLA-B*5701 should be excluded prior to prescribing ABC, especially in patients who are not of African descent.

#### Zidovudine

We now recommend reserving AZT for use only in special circumstances in first-line therapy. If both TDF and ABC are unavailable or contraindicated, then AZT should be used, provided that the Hb is > 8 g/dL.

### Additional syndromes related to nucleoside reverse transcriptase inhibitors

#### Haematological toxicity

Cytopenias occur commonly in patients with HIV infection without exposure to ART. Patients receiving AZT or cotrimoxazole (CTX) may experience full blood count (FBC) abnormalities. Zidovudine can cause anaemia and neutropenia; platelet counts generally rise with the use of the drug. Monitoring is necessary with AZT (see Table 2). It is unusual, however, to see haematological toxicity develop after 6 months. Macrocytosis is usual with AZT therapy and is of little consequence. Routine measurement of vitamin B12 and folate concentrations is not needed.

◦ **Common pitfall: Discontinuing AZT because of macrocytosis.** This is of little consequence and does not necessarily portend subsequent anaemia.

Pure red cell aplasia, which presents with severe anaemia and a low reticulocyte production index, has rarely been associated with 3TC and FTC.^9,10^ A bone marrow examination should be performed to confirm the condition. A polymerase chain reaction (PCR) test should be conducted to exclude the presence of parvovirus B19 infection. If 3TC and FTC are contraindicated because of pure red cell aplasia, then we suggest contacting an expert for advice about alternative regimens.

#### Hyperlactataemia and lactic acidosis

Lactic acidosis is a rare but serious and potentially fatal side effect of NRTIs, most commonly associated with stavudine (d4T), particularly when combined with didanosine (ddI). These drugs are no longer used. It can also occur occasionally with AZT. Symptomatic hyperlactatemia without acidosis is more common and is associated with the same drugs. Neither lactic acidosis nor hyperlactatemia without acidosis is seen with the newer, safer NRTIs such as TDF, ABC, 3TC or FTC. Symptoms are non-specific and include nausea and vomiting, abdominal pain, dyspnoea, fatigue and weight loss. A raised lactate (> 5 mmol/L) together with metabolic acidosis confirms the diagnosis of lactic acidosis. Low serum bicarbonate (< 20 mmol/L) is the most sensitive marker of acidosis. Patients receiving AZT who develop hyperlactatemia should be switched to alternative drugs, and lactate should be monitored serially until resolution. In severe patients, admission may be required.

#### Lipoatrophy

The thymidine analogue NRTIs (AZT and especially d4T) are associated with subcutaneous fat loss (most noticeable in the face, limbs and buttocks). Lipoatrophy improves when d4T/AZT are substituted with TDF or ABC, but resolution is very slow and often incomplete; therefore, it is important to recognise lipoatrophy early or, better still, to use NRTIs that are not associated with the condition. Although d4T is no longer used, patients who received it historically may still have lipoatrophy.

## 3. Integrase strand transfer inhibitor class of antiretroviral drugs

### Key points

➢ Two integrase strand transfer inhibitors (InSTIs) are available in southern Africa, namely, DTG and raltegravir (RAL).
➢ Dolutegravir is preferred to RAL because it has a higher barrier to resistance, is available in FDC formulation and can be taken once daily.
➢ Dolutegravir has been shown to have greater efficacy than efavirenz (EFV), driven largely by superior tolerability.
➢ Dolutegravir causes a small increase in serum creatinine (usually 10 mmol/L – 20 mmol/L) because of interference with tubular creatinine secretion; however, this does not represent a decline in renal function.
➢ Although definitive data are still lacking, DTG may be teratogenic in a small proportion of patients; thus, treatment decisions in women of reproductive age should be discussed and evaluated carefully (see section 19).
➢ Weight gain is a newly recognised side effect of InSTIs, more so with DTG than with RAL, and more so in black women and in patients with lower baseline CD4^+^ counts and higher VLs.

### Overview of integrase strand transfer inhibitors

Integrase strand transfer inhibitors – often simply termed ‘integrase inhibitors’ – work by preventing the transfer of proviral DNA strands into the host chromosomal DNA. Currently, two InSTIs are available in southern Africa: DTG and RAL. Dolutegravir is preferred to RAL because of its higher barrier to resistance, its availability in FDC formulation and the ability to take the drug once daily. The SPRING-2 trial compared DTG- and RAL-containing first-line regimens and found no significant differences in virological suppression, and adverse effects were similar between treatment groups^11^; however, although no patients in the DTG arm were found to have developed resistance, one patient in the RAL arm developed InSTI resistance and four developed NRTI resistance. The high barrier to resistance of DTG-containing ART regimens has been replicated in other first-line studies and in a study of ART-experienced patients in which DTG was compared with RAL.^12,13,14^ In a meta-analysis that included clinical trials and observational studies, the emergence of InSTI resistance was more common with RAL than with DTG (3.9% vs. 0.1%).^15^ However, the emergence of InSTI resistance in patients receiving RAL can compromise second-generation InSTIs, such as DTG.

Dolutegravir use has been shown to be superior to EFV-based ART in the SINGLE trial.^12^ This difference was largely driven by the superior tolerability of the DTG arm: 2% in the DTG arm compared with 10% in the EFV arm had an adverse event leading to discontinuation of the study drug. Dolutegravir showed superior rates of viral suppression compared with EFV (71% vs. 63% at 144 weeks).

Dolutegravir-based regimens have also been shown to be superior to protease inhibitor (PI)-based regimens. As a first-line therapy, DTG was superior to darunavir/ritonavir (DRV/r) in terms of both viral suppression rates and side effect profile.^13^ The ARIA trial of ART-naive women demonstrated DTG’s non-inferiority to atazanavir (ATV)/ritonavir (ATV/r), although with a statistically significantly higher rate of viral suppression and fewer side effects overall.^16^ In the DAWNING trial considering second-line regimens, DTG was found to be superior to lopinavir/ritonavir (LPV/r).^17^ Importantly, at least one fully active NRTI was genotypically confirmed at baseline in this trial.

Data from the Tsepamo surveillance study in Botswana demonstrated a statistically higher rate of neural-tube defects (NTDs) amongst women who were taking DTG at the time of conception (0.3% vs. 0.1% in women receiving other ARTs in the periconception period).^18^ Unlike in South Africa, folate fortification of staple foods does not occur in Botswana. In contrast to the Botswana data, no NTDs were reported in a Brazilian cohort of 1468 women, 382 of whom were DTG-exposed.^19^ Although additional data will undoubtedly be forthcoming, it should be noted that the absolute risk is < 0.5%, which may be outweighed by the additional benefits of DTG over alternative therapies. We recommend that women of childbearing potential (WOCP), particularly those who wish to become pregnant or who have no reliable access to effective contraception, should be counselled adequately about the potential risks and benefits of DTG- versus EFV-based ART and should be offered a choice of first-line regimens.

### Common side effects

Dolutegravir and RAL are generally well tolerated, with most side effects being mild and very rarely leading to discontinuation. Dolutegravir may cause a mild increase in serum creatinine because of interference with tubular secretion. This does not represent renal damage and is not an indication of switching to another drug. The rise in creatinine occurs within the first few weeks and persists for as long as the patient remains on DTG.

◦ **Common pitfall: Assuming that the rise in creatinine seen in patients on DTG necessarily represents renal failure.** In reality, the effect of DTG on creatinine secretion is of no consequence and does not represent a decline in renal function.

Raltegravir and DTG can cause headaches when started, but this usually resolves after sometime. These drugs may also cause insomnia and neuropsychiatric side effects. Raltegravir and DTG can occasionally cause hypersensitivity rashes, including life-threatening rashes. Weight gain is more pronounced in patients taking an InSTI as part of their ART regimen (with the exception of cabotegravir, which is not currently available in South Africa). Black women, patients with low baseline CD4^+^ counts and patients with high baseline VLs appear to be at greatest risk.^20^ The risk also appears to be moderated by the companion drugs in the patient’s ART regimen. In the ADVANCE trial, women on a tenofovir alafenamide (TAF) + FTC + DTG regimen were found to gain a median of 10 kg over 96 weeks, with little evidence of a plateau in the increase.^21^ In women, median weight gain in the same period in the TDF + FTC + DTG arm was 5 kg, and 3 kg in the TDF + FTC + EFV arm. In men, weight gain was approximately half as much in each arm. The long-term health implications of these findings are currently unclear; however, clinicians should be aware of the possibility of weight gain and encourage appropriate exercise and dietary measures to limit this.

Dosage and common adverse drug reactions (ADRs) of InSTIs are described in Table 3.

**TABLE 3:** Dosage and common adverse drug reactions of integrase strand transfer inhibitors available in southern Africa.

Drug	Recommended dosage	Common or severe ADR
RAL	400 mg 12 hourly	Headache and other CNS side effects, gastrointestinal upset, hepatitis and rash (rare), rhabdomyolysis (rare). Weight gain.
DTG	50 mg daily	Insomnia, headache and other CNS side effects, gastrointestinal upset, hepatitis and rash (rare). Possibly **teratogenic**. Weight gain.

ADR, adverse drug reaction; CNS, central nervous system; DTG, dolutegravir; RAL, raltegravir.

### Key drug–drug interactions with dolutegravir

Key drug–drug interactions involving DTG are summarised in Table 4.

◦ **Common pitfall: Forgetting to dose DTG twice daily when RIF-based tuberculosis treatment is commenced.**

**TABLE 4:** Key drug–drug interactions with dolutegravir.

Drug	Action required
RIF	Administer DTG twice daily (i.e. 50 mg 12 hourly) until 2 weeks after stopping RIF.
Metformin	Do not exceed metformin 500 mg 12 hourly.
Carbamazepine, phenytoin	Give alternative anticonvulsant if possible (e.g. lamotrigine or topiramate). If carbamazepine is used, then administer DTG 12 hourly. Avoid phenytoin with DTG altogether.
Polyvalent cation-containing agents (e.g. antacids, laxatives, sucralfate, iron and calcium supplements)	For magnesium-/aluminium-containing antacids, administer > 2 h after or > 6 h before DTG dose. For iron/calcium supplements, either take with food or apply intervals above.
Etravirine	Do not use DTG + etravirine together unless a boosted PI is also used in the combination.

DTG, dolutegravir; RIF, rifampicin; PI, Protease inhibitors.

## 4. Non-nucleoside reverse transcriptase inhibitor class of antiretroviral drugs

### Key points

➢ Efavirenz remains a good first-line ART option for patients who tolerate DTG poorly, or where DTG is contraindicated or declined.
➢ Efavirenz 400 mg is not inferior to EFV 600 mg and offers a somewhat improved side-effect profile. However, it is currently not available in FDC and has not been well studied in patients receiving rifampicin (RIF)-based TB treatment or in pregnant women.
➢ Rilpivirine (RPV) is another good first-line option, but it is not available in FDC, cannot be co-administered with RIF-based TB treatment and should not be started in patients with a VL > 100 000 copies/mL.
➢ Nevirapine (NVP) is no longer recommended for new patients because of its adverse side effect profile.
➢ Etravirine (ETR) may be used as part of third-line therapy where appropriate, but is not recommended as a first-line agent.

### Overview of non-nucleoside reverse transcriptase inhibitors

Non-nucleoside reverse transcriptase inhibitors work by binding irreversibly to HIV’s reverse transcriptase enzyme, which causes a conformational change in the enzyme’s active site and impairs its functioning. The four NNRTIs currently available in southern Africa are EFV, NVP, RPV and ETR.

### Individual non-nucleoside reverse transcriptase inhibitors

#### Efavirenz

**Efavirenz is available in 600 mg and 400 mg formulations:** Efavirenz 600 mg is available in public sector programmes in most countries in southern Africa. There is extensive clinical experience with the formulation, and it is available in FDC. Efavirenz 400 mg showed non-inferior efficacy with moderately improved tolerability in the ENCORE1 study.^22^ However, there are only limited pharmacokinetics data in pregnant patients, and in patients receiving RIF-based TB treatment. Efavirenz 400 mg is currently also not available in FDC. For these reasons, we do not recommend the routine use of EFV 400 mg in first-line ART. It remains an appropriate choice, however, in selected patients.

Efavirenz frequently causes neuropsychiatric effects in the first few weeks of therapy, typically presenting with insomnia, vivid dreams and dizziness. Both dysphoria and euphoria may occur. Patients starting on EFV should be warned about these symptoms and should be reassured that the symptoms usually resolve within the first few weeks, and if not, then an alternative can be substituted. Psychosis may occasionally occur. If the neuropsychiatric effects of EFV are not tolerated, then the patient should be switched to RPV, DTG or lower-dose EFV. Recently, a late-onset encephalopathy syndrome has been linked to EFV.^23^ This is characterised by a subacute encephalopathy and cerebellar dysfunction, frequently presenting months to years after commencing EFV, and is associated with supratherapeutic EFV levels. Patients who are genetically slow metabolisers of EFV may be predisposed to this syndrome. Two common CYP2B6 polymorphisms linked to slow EFV metabolism have been shown to occur with increased frequency in patients of African descent.^24^ This predisposition to toxic EFV levels may be further exacerbated in patients of low body weight and in those taking concomitant isoniazid, which inhibits an accessory EFV metabolism pathway via CYP2A6. Patients with a compatible clinical syndrome, in the absence of an alternative cause, should have plasma EFV levels measured and should be switched to a non-EFV-based regimen. Clinical improvement is typically seen within 10–21 days after stopping EFV.

Efavirenz may also cause a drug-induced hepatitis. A subset of these cases appears to occur relatively late, several months or even years after the drug has been initiated.^25^ It is important that this diagnosis is considered in the differential diagnosis of a subacute hepatitis syndrome. Gynaecomastia can occur with the use of EFV.^26^ This is not related to lipodystrophy. The onset occurs several months after initiation of ART and it may be bilateral or unilateral. The mechanism appears to be related to oestrogen receptor activation in breast tissue by EFV.^27^ It is important to exclude other common causes of gynaecomastia, such as other medications (including spironolactone, calcium channel blockers and metoclopramide). A serum testosterone test is useful in excluding hypogonadism as a possible cause. If serum testosterone is low, then other appropriate investigations should be carried out to identify the cause and manage accordingly; if serum testosterone is normal, then EFV should be substituted, bearing in mind the general principles of single-drug substitutions (patients who are virologically suppressed should be switched to DTG or RPV). Resolution of gynaecomastia is generally slow, taking months, and may be incomplete in a small percentage of patients.^28^ It is therefore important to manage the expectations of the patient in this regard.

#### Rilpivirine

**Another option in first-line ART is RPV, a second-generation NNRTI:** Rilpivirine is inexpensive, but not currently available in FDC in the region. An important drawback is that it should not be started in a patient with a VL > 100 000 copies/mL, as it is inferior to EFV in such patients.^29^ Rilpivirine has a lower incidence of neuropsychiatric side effects and rashes than EFV.^30^ There are several important drug–drug interactions with RPV. Amongst other considerations, RPV cannot be co-administered with RIF or proton pump inhibitors (PPIs). Histamine-2-receptor antagonists need to be administered 12 h before or 4 h after taking RPV. Rilpivirine should be taken with food to increase absorption.

◦ **Common pitfall: Prescribing RPV without first checking baseline VL.** Rilpivirine is less efficacious than comparator drugs when VL is > 100 000 copies/mL.

#### Nevirapine

**We no longer recommend NVP use for new patients starting ART because of the severe toxicity that may be associated with its use:** In patients currently tolerating NVP, there is no reason to switch treatment because of toxicity concerns, as toxicity characteristically occurs in the first 3 months of NVP treatment and not later. However, switching for the purpose of simplification to a once-daily regimen should be considered, provided that there is virological suppression.

#### Etravirine

**Etravirine is a second-generation NNRTI that has been studied in treatment-experienced patients rather than in ART-naive patients:** As seen with RPV, the activity of ETR is not affected by the first-generation NNRTI’s signature K103N resistance mutation.

### Hypersensitivity with non-nucleoside reverse transcriptase inhibitors

Rash is common with NNRTIs in the first 6 weeks of therapy, notably more severely and frequently with NVP. If the rash is accompanied by systemic features (e.g. fever, elevated alanine transaminase [ALT] or hepatitis), mucosal involvement or blistering, then the NNRTI should be discontinued immediately and re-challenge must not be performed as these are features of life-threatening reactions. If the rash is mild and occurs without these features, then the NNRTI can be continued and the rash can be treated symptomatically with antihistamines and possibly topical steroids. Systemic steroids should not be used. If there is a severe reaction to EFV or NVP, then we do not recommend switching to RPV or ETR – rather use DTG or a PI.

Dosage and common ADRs of NNRTIs available in southern Africa are described in Table 5.

◦ **Common pitfall: Immediately discontinuing NNRTIs in the case of a mild rash without systemic features.** Such rashes often resolve if treatment is continued, although close monitoring is required.

**TABLE 5:** Dosage and common adverse drug reactions of non-nucleoside reverse transcriptase inhibitors available in southern Africa.

Drug	Recommended dosage	Common or severe ADR†
EFV	600 mg at night (400 mg at night if the patient is < 40 kg). A dose of 400 mg can also be used in patients > 40 kg.	CNS symptoms (vivid dreams, problems with concentration, dizziness, confusion, mood disturbance, psychosis, **late-onset encephalopathy**), **rash, hepatitis** gynaecomastia
NVP	200 mg daily for 14 days and then 200 mg 12 hourly	**Rash, hepatitis**
RPV	25 mg daily with food	**Rash, hepatitis**, CNS symptoms (all uncommon)
ETR‡	200 mg 12 hourly	**Rash** and **hepatitis** (both uncommon)

ADR, adverse drug reaction; CNS, central nervous system; EFV, efavirenz; ETR, etravirine; NNRTI, non-nucleoside reverse transcriptase inhibitors; NVP, nevirapine; RPV, rilpivirine.

† , Life-threatening reactions are indicated in **bold**.

‡ , NNRTI combinations to be avoided include (1) ETR + ATV/r (because of drug interaction) and (2) ETR + DTG unless a boosted PI is also used in the combination (because of drug interaction).

## 5. Protease inhibitor class of antiretroviral drugs

### Key points

➢ Three PI combinations are recommended in southern Africa: lopinavir (LPV), ATV or darunavir (DRV), each given with low-dose ritonavir (RTV, indicated as /r) for pharmacokinetic boosting.
➢ Ritonavir-boosted lopinavir is the only PI combination that can be used with RIF-based TB treatment, but the dose of LPV/r must be doubled.
➢ Atazanavir and DRV offer a better side effect profile than LPV.
➢ Darunavir has the highest barrier to resistance of any drug in this class.

### Overview of protease inhibitors

Protease inhibitors are a class of agents that inhibit HIV’s protease enzyme, which is required to cleave HIV’s polyproteins into the final protein products that permit the production of infectious viral particles. Inhibition of this process results in immature, non-infectious virions.

Three PI combinations are recommended for use in southern Africa: LPV, ATV and DRV, each given with low-dose ritonavir.

Ritonavir is a PI in its own right, but is used principally as a pharmacokinetic ‘booster’. As a potent inhibitor of CYP3A4, its use results in higher drug levels and prolonged half-lives of its companion PI. This allows for lower or less frequent PI dosing and decreases the chances of developing viral resistance. In rare situations, ATV is used without boosting in first-line therapy. However, this inhibition of CYP3A4, together with several other cytochrome P450 (CYP) enzymes and p-glycoprotein, results in numerous drug–drug interactions with other medications (see section 17).

◦ **Common pitfall: Not using a drug interaction checker when prescribing PI-based ART with other medications.** Clinically relevant drug–drug interactions are common with this class.

All PIs may be associated with cardiac conduction abnormalities (especially PR interval prolongation). This seldom results in clinically significant effects, but caution should be taken when co-prescribing other drugs that cause delayed cardiac conduction, such as macrolides or bedaquiline. All PIs are, to some extent, associated with metabolic side effects. Elevated triglycerides (TGs) and elevated low-density lipoprotein cholesterol (LDL-C) are class effects, although these side effects are more pronounced with LPV/r than with other PI combinations.^31,32^

Dosing and common ADRs of PIs are presented in Table 6.

**TABLE 6:** Dosage and common adverse drug reactions of protease inhibitor drugs available in southern Africa.

Drug/combination	Recommended dosage	Common or severe ADR
ATV/r†	ATV/r 300 mg/100 mg daily If given with EFV: ATV/r 400/100 mg daily	Unconjugated hyperbilirubinaemia (visible jaundice in a minority of patients), dyslipidaemia (low potential), renal stones (rare) and hepatitis (uncommon)
LPV/r	400 mg/100 mg 12 hourly or 800 mg/200 mg daily (only if PI-naïve)	Gastrointestinal upset, dyslipidaemia and hepatitis
DRV/r	600 mg/100 mg 12 hourly 800 mg/100 mg daily (only if no DRV mutations) Consider 400 mg/100 mg daily (only if no DRV mutations)	Gastrointestinal upset, rash, dyslipidaemia, hepatitis (uncommon). Contains sulphonamide moiety (use with caution in patients with sulpha allergy)

ADR, adverse drug reaction; ATV/r, atazanavir/ritonavir; DRV/r, darunavir/ritonavir; EFV, efavirenz; LPV/r, lopinavir/ritonavir; PI, protease inhibitor.

† , Avoid the combination of ETR + ATV/r (because of drug interaction).

### Individual protease inhibitors

#### Lopinavir

**Lopinavir is co-formulated with ritonavir (e.g. Aluvia):** In general, this twice-daily regimen has greater gastrointestinal (GI) side effects than other PI combinations, and is associated with a worse metabolic profile. Lopinavir is the only PI that can be used concurrently with RIF-based TB treatment; the LPV/r dose has to be doubled in this instance to 800 mg/200 mg twice daily until 2 weeks after RIF has been stopped (see section 18).

◦ **Common pitfall: Forgetting to double the dose of LPV/r when starting RIF-based TB treatment.**

#### Atazanavir

**Atazanavir is generally better tolerated than LPV and can be taken once daily:** It has important drug interactions with drugs that reduce stomach acidity, such as PPIs. Atazanavir may cause an *unconjugated hyperbilirubinaemia* as a result of inhibition of the hepatic enzyme Uridine 5'-diphospho-glucuronosyltransferase. Although the hyperbilirubinaemia is harmless and does not reflect a drug-induced liver injury (DILI), a minority of patients will become visibly jaundiced, and this may require changing ART regimens for cosmetic reasons.

◦ **Common pitfall: Mistaking the unconjugated hyperbilirubinaemia sometimes seen with ATV use with a DILI.** Conversely, it is equally important to note that ARVs can also cause a true DILI, and therefore a complete liver function test (LFT) panel should be performed to distinguish between the two possibilities.

#### Darunavir

**Darunavir has the highest barrier to resistance of any PI:** Mutations selected by ATV or LPV can compromise DRV efficiency. For patients with mutations that confer any degree of resistance to DRV (e.g. I50V, L76V and I84V), the dose should be DRV/r 600 mg/100 mg twice daily. For patients without any DRV mutations, the drug can be taken at a dose of DRV/r 800 mg/100 mg once daily. There is evidence, however, that DRV/r 400 mg/100 mg once daily may be sufficient in this scenario, especially for patients with suppressed VLs at the time of the switch.^33,34^ Compared with a twice-daily dosing, a once-daily dosing offers the benefits of reduced pill burden and better side effect profile. As with ATV, DRV cannot be co-prescribed with RIF-based TB treatment.

◦ **Common pitfall: Prescribing ATV or DRV in patients receiving RIF-based TB treatment.** Lopinavir/ritonavir is the only PI combination that can be co-prescribed safely with RIF, but the dose of LPV/r must be adjusted as above.

## 6. Initiation and timing of antiretroviral therapy

### Key points

➢ All individuals diagnosed with HIV should be initiated on ART.
➢ Delays to start ART should be minimised. Several studies have demonstrated that it is safe to initiate.
➢ ART on the same day as diagnosis or on receipt of CD4^+^ count result, with the main benefit being improved retention in care.
➢ Screening for TB, cryptococcal meningitis (CM) and other OIs prior to ART initiation is important, as these conditions may necessitate delaying ART initiation.

### Overview

All patients who are diagnosed with HIV should be initiated on ART as soon as possible. Exceptions include patients presenting with CM or tuberculosis meningitis (TBM) – see below.

#### Benefits of antiretroviral therapy in reducing morbidity and mortality

With ART-induced viral suppression, the CD4^+^ lymphocyte count usually increases, which is accompanied by a restoration of pathogen-specific immune function. For most patients, this results in a dramatic reduction in the risk of HIV-associated morbidity and mortality. For patients who start ART with preserved CD4^+^ counts, ART is able to prevent the decline in CD4^+^ count observed in untreated patients and thereby prevent clinical complications of HIV infection. The benefits in morbidity and mortality extend to patients with relatively preserved CD4^+^ counts. The START and TEMPRANO ANRS 12136 trials showed significant individual clinical benefits when starting ART immediately in patients with CD4^+^ counts > 500 cells/µL rather than deferring until a certain lower CD4^+^ threshold or clinical indication was met.^35,36^

#### Benefits of antiretroviral therapy in reducing transmission

The HPTN 052 trial showed that treating the HIV-positive partner in a serodiscordant relationship with ART was associated with a 93% reduction in transmission risk to the uninfected partner, with the only linked transmissions occurring from partners without a suppressed VL.^37^ Further evidences in serodiscordant couples from the PARTNER, PARTNER2 and Opposites Attract trials have confirmed that HIV is essentially not transmittable when the VL is suppressed.^38,39,40^ Community-level evidence has also demonstrated a reduction in HIV incidence as ART rollout is scaled up. Therefore, early ART initiation has significant public health benefits.

#### Antiretroviral therapy in primary human immunodeficiency virus infection

In patients who are diagnosed with HIV during *acute seroconversion*, we advise counselling and initiating ART as soon as possible. Expedited ART initiation is preferable as there is evidence that this may limit the size of the HIV reservoir.^41^ Once the patient is established on ART, additional counselling may be required for patients who start ART in this acute stage because there is limited time for extensive pre-ART counselling, and there is often considerable psychological distress around this time.

### Antiretroviral therapy initiation in ‘elite controllers’

A minority of patients (< 1%) have very effective immune control of HIV infection and can control HIV viraemia at undetectable levels even in the absence of ART; these patients are termed ‘elite controllers’.

Although definitive data are lacking for this patient subgroup, we advise initiating ART in elite controllers too, as indirect evidence suggests a potential benefit. Elite controllers still have evidence of chronic immune activation and inflammation that may drive non-infectious morbidities.^42^ Elite controllers have also been shown to have a higher rate of hospitalisation than patients who are virologically controlled by ART.^43^ Furthermore, a prospective study of HIV-positive ‘controllers’, who were able to control viral replication to < 500 copies/mL, showed that HIV therapy led to improvements in markers of immune activation and immune exhaustion, and a slightly improved self-reported quality of life.^44^ This trial included elite controllers.

One important consideration in such patients is that careful attention should be given to confirm the diagnosis of HIV before starting ART. These patients typically have a positive HIV enzyme-linked immunosorbent assay (ELISA) test, undetectable HIV VL, CD4^+^ count in the normal range and are clinically well. The possibility of a false-positive HIV ELISA test should be excluded either by qualitative HIV DNA PCR or Western Blot assay. If the patient previously had a detectable HIV VL, then this would also serve as confirmation. Such patients may need to be discussed with a laboratory virologist to assist with confirmation of HIV status.

◦ **Common pitfall: Not confirming the HIV status of an ‘elite controller’.** If such patients have been diagnosed with HIV based on an HIV ELISA or rapid detection test, then confirmation of their HIV status should be sought by additional testing methods to exclude the possibility of a false-positive result.

#### Commencing antiretroviral therapy at the first clinic visit

Several studies have demonstrated that it is possible to initiate ART safely on the same day as HIV diagnosis or reporting of the CD4^+^ count result.^45,46,47^ These studies have demonstrated less overall loss to follow-up when ART is initiated immediately in selected patients. Now that treatment is recommended irrespective of CD4^+^ count, this same-day strategy should be considered as a means to improve retention in care.

When deciding to initiate ART on the same day as diagnosis, considerations should include the following:

- The patient should be motivated to start immediately.
- Same-day initiation is not an adherence support ‘short cut’; ongoing support can occur in the days and weeks immediately after initiation.
- Patients starting TDF (who are the majority) should be contactable in the event of a CrCl < 50 mL/min and advised to return to the clinic immediately.
- A serum/plasma cryptococcal antigen (CrAg) test should be performed in patients with a CD4^+^ count < 200 cells/µL; again, the patient should be contactable in the event of a positive result and must be advised to return to the clinic immediately.
- Symptom screen for TB and CM before initiation of treatment remains important, and a positive screening requires further investigation prior to ART initiation.

#### Medical reasons to delay antiretroviral therapy initiation

Medical reasons to delay ART initiation are outlined in Table 7.

**TABLE 7:** Medical reasons to delay antiretroviral therapy initiation.

Reason	Action
Diagnosis of CM	Defer ART for 4–6 weeks after start of antifungal treatment.
Diagnosis of TBM or tuberculoma	Defer ART until 4–8 weeks after start of TB treatment.
Diagnosis of TB at non-neurological site	Defer ART up to 2 weeks after start of TB treatment if CD4^+^ ≤ 50 cells/µL and up to 8 weeks if CD4^+^ > 50 cells/µL.
Headache	Investigate for meningitis before starting ART.
TB symptoms (cough, night sweats, fever and recent weight loss)	Investigate for TB before starting ART.
Significantly abnormal LFTs (ALT > 200 U/L or jaundice)	Investigate and address the cause before starting ART, including other drugs causing DILI.

ART, antiretroviral therapy; ALT, alanine transaminase; CM, cryptococcal meningitis; DILI, drug-induced liver injury; LFTs, liver function tests; TB, tuberculosis; TBM, tuberculosis meningitis.

Tuberculosis: Decisions regarding the timing of ART in patients with TB should generally be based on the CD4^+^ count.

- **CD4**^+^ **count ≤ 50 cells/µL:** Antiretroviral therapy should be regarded as urgent, with the aim to start therapy within 2 weeks following the commencement of TB treatment. A meta-analysis of RCTs has demonstrated that this approach reduces mortality.^48^ It is advised to commence ART after it is clear that the patient’s TB symptoms are improving and that TB therapy is tolerated. The exception to this is the case of CM or TBM (see below).
- **CD4**^+^ **count > 50 cells/µL:** Antiretroviral therapy can be delayed until 8 weeks after starting TB treatment, but no later. However, if the patient has other World Health Organization (WHO) stage 4 conditions, then ART should be initiated 2 weeks after TB treatment is started. The exception to this is CM or TBM. The longer delay before commencing ART in this group is anticipated to reduce the risk of IRIS (see section 26). The aforementioned meta-analysis of RCTs did not show a higher risk of acquired immune deficiency syndrome (AIDS) progression/mortality in this group when ART initiation was delayed until approximately 8 weeks after starting TB treatment, but a reduced risk of TB-IRIS.^48^

**Tuberculosis meningitis:** Patients with TBM are an exception to the above: starting ART immediately or at 2 months following the diagnosis was shown to have similar high mortality, with more complications in the immediate group.^49^ We recommend starting ART 4–8 weeks after TBM diagnosis.

There are important drug interactions and shared side effects when ART is co-administered with TB therapy (see section 18). When ART is commenced, patients should be warned that TB symptoms or signs may temporarily worsen and new features may occur in the first 3 months as a result of TB-IRIS (see section 26).

**Cryptococcal disease:** For patients with CM, the optimal time to start ART is 4–6 weeks from the time of starting CM treatment. The Cryptococcal Optimal ART Timing (COAT) trial demonstrated significantly higher mortality in patients who started ART in hospital 1–2 weeks after CM diagnosis than in those starting 5–6 weeks after diagnosis.^50^

For patients diagnosed with cryptococcal antigenaemia who have CM excluded by lumbar puncture (LP), ART can be commenced immediately.

Patients commenced on ART prior to a positive reflex CrAg result should be referred immediately for LP to exclude CM. In patients with a negative cerebrospinal fluid (CSF) CrAg result (i.e. CM is excluded), ART can be continued and fluconazole pre-emptive therapy should be initiated. It is unclear, however, whether to interrupt ART in patients with a positive CSF CrAg result.

For further details, refer to the 2019 Southern African HIV Clinicians Society guidelines for the prevention, diagnosis and management of cryptococcal disease amongst HIV-infected persons.

**Starting antiretroviral therapy in patients with other opportunistic infections and acute illnesses:** In the case of most OIs and acute illnesses (e.g. pneumocystis or bacterial pneumonia), the aim should be to initiate ART within 2 weeks of commencing treatment for that infection.^51^ In patients with severe Kaposi’s sarcoma and lymphoma, ART counselling should be expedited and ART should be initiated as soon as possible.

In HIV-infected patients admitted to hospital and unable to take oral medications, for example, patients in intensive care unit (ICU):

- If the patient is receiving ART, then this should be continued – through nasogastric tube (NGT) if necessary – and only interrupted if the GI tract is not functional (e.g. ileus).
- If the patient is not yet received ART, then it should not be commenced if the reason for admission is an acute critical illness or injury. There are several potential problems associated with commencing ART in this setting: lack of adequate counselling, GI dysfunction, malabsorption and possible development of resistance.
- There are no intravenous options for ART. In patients admitted to the ICU for prolonged periods, ART initiation in the unit should be considered after multi-organ failure has resolved. Certain ART preparations should not be administered via NGT. In general, paediatric syrups can be administered via NGT. A pharmacist should always be consulted regarding which ART drugs can be administered via NGT and how to do this.

## 7. Baseline investigations

### Confirming the diagnosis of human immunodeficiency virus

Prior to the initiation of lifelong ART, it is recommended that HIV infection is confirmed with two different testing methods, at least one of which should be a laboratory-based test. Acceptable combinations include the following:

- rapid detection test + ELISA
- rapid detection test + VL
- ELISA + VL.

Note that a VL may be undetectable in < 1% of patients not receiving ART, that is, ‘elite controllers’.

### Baseline investigations

Baseline investigations for ART are summarised in Table 8.

**TABLE 8:** Summary of baseline investigations for antiretroviral therapy.

Investigation	Comment
CD4^+^ count	If CD4^+^ count < 200 cells/μL, then CPT is required and sCrAg testing needs to be performed.
Baseline VL	Can also serve as a confirmatory HIV test.
ALT	If raised, then will need workup and may influence ART regimen choice.
Creatinine	Avoid TDF if CrCl < 50 mL/min. Other NRTIs except ABC require dose adjustment if CrCl < 50 mL/min.
HBsAg	See section 20.
Syphilis serology	
sCrAg	Only required in patients with a CD4^+^ count < 200 cells/μL. If sCrAg-positive, exclude CM by LP. See the section on CM management (section 27) for further details.

ABC, abacavir; ALT, alanine transaminase; CrCl, creatinine clearance rate; CM, cryptococcal meningitis; CPT, cotrimoxazole preventive therapy; HBsAg, hepatitis B surface antigen; LP, lumbar puncture; sCrAg, serum/plasma cryptococcal antigen; TDF, tenofovir disoproxol fumarate; VL, viral load; HIV, human immunodeficiency virus; ART, antiretroviral therapy; NRTI, nucleoside reverse transcriptase inhibitor.

### Symptom screen

We also advise a symptom screen for:

- **Tuberculosis:** patients should be asked about cough, weight loss, fever, night sweats and a possible TB contact. If any of these symptoms are present, then sputum should be sent for Xpert analysis, and if hospitalised or the CD4^+^ count is < 200 cells/µL, a urine lipoarabinomannan (LAM) assay should be performed.
- **Cryptococcal meningitis:** patients should be asked about new onset of headache; serum cryptococcal antigen (sCrAg) testing and possibly an LP should be performed if this symptom is present.

If the patient’s symptom screen is positive, then ART should be deferred until the results of the Xpert, LAM, sCrAg test or LP (as indicated) are known. Delays in this process should, however, be kept to a minimum.

## 8. Viral load

Viral load monitoring is key to the success of ART. Decisions to change ART made on the basis of virological failure, rather than on clinical or immunological failure alone, have been shown to result in better patient outcomes.^52^ If the VL is undetectable, then the virus cannot mutate and develop resistance. A sustained VL < 50 copies/mL is associated with the most durable benefit. A suppressed VL also prevents the transmission of HIV to contacts.

### Timing of viral load monitoring in the patient starting antiretroviral therapy (Figure 1)

We recommend a *baseline VL* for the following reasons:

1. The 3-month VL can then be compared with the baseline VL to detect > 2 log_10_ drop, and if this has not occurred, then it allows for early adherence intervention.
2. It may guide NNRTI selection (RPV should not be used if VL > 100 000 copies/mL).
3. It confirms the diagnosis of HIV (antibody tests may very rarely give a false-positive result).

**FIGURE 1:**
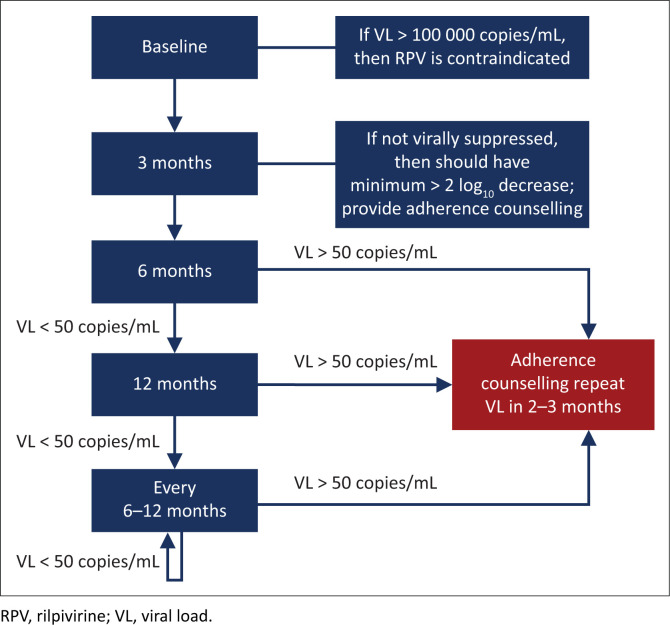
Timing of viral load monitoring of the patient starting antiretroviral therapy. For patients with a viral load > 50 copies/mL on two consecutive occasions, refer to the text.

A *3-month VL* is desirable to detect adherence problems early before resistance develops. A subset of patients who start ART with a very high VL may not be fully suppressed at 3 months despite 100% adherence, but such patients would have had a > 2 log_10_ drop in VL from baseline if adherence is optimal and there is no resistance. Therefore, the 3-month result should be interpreted in relation to the baseline VL. All patients who have a detectable VL at 3 months should receive additional adherence interventions. In general, a patient’s VL declines very fast on InSTI-based regimens.

If the 3-month VL is undetectable, then VL monitoring is recommended at 6 months and every 6 months thereafter. In patients who have an undetectable VL for more than 12 months, and who demonstrate reliable adherence and follow-up, it may be acceptable to reduce the frequency of VL monitoring to 12 monthly.

**If the VL is > 50 copies/mL at any stage, then this should be an indication for urgent action:** The patient should receive counselling and interventions should be implemented to improve adherence. A repeat measurement of VL should then be done in 2–3 months.

### Interpreting viral load results

#### Virological criteria for treatment success

Treatment success is defined as a decline in VL to < 50 copies/mL within 6 months of commencing ART, and sustained thereafter.

#### Virological criteria for treatment failure

**Treatment failure is defined as a confirmed VL > 50 copies/mL on two consecutive measurements taken 2–3 months apart:** The decision to alter ART should therefore be based on the results of repeat testing after 2–3 months, following intensive adherence counselling*. Although previous guidelines used a threshold of 1000 copies/mL to define virological failure, there is now good evidence that a VL > 50 copies/mL is robustly associated with subsequent virological failure*, although this has not been established.^53,54^ Sustained viral replication, even at these low levels, can lead to the accumulation of resistance mutations (although this has not yet been definitively established in the case of DTG).

#### Viral blips

Isolated detectable HIV VLs < 1000 copies/mL, followed by an undetectable VL, are termed ‘viral blips’ and alone are not a reason to change the ART regimen.

- Viral blips can be caused by immune activation (such as from an acute infection), variability in the laboratory testing thresholds or intermittent poor adherence. Provided that they are infrequent, and the VL returns to being undetectable at the next measurement, they are not regarded as consequential.

#### Reasons for a high viral load

A high VL can be attributed to one or more of these three factors:

- inadequate patient adherence (most commonly)
- resistance to the prescribed ART – including both acquired and transmitted drug resistance
- inadequate ART drug levels as a result of altered pharmacokinetics, such as absorption difficulties, or drug–drug interactions.

These explanations are not mutually exclusive. For instance, inadequate patient adherence frequently leads to the development of resistance in patients on a non-DTG-containing regimen.

*Transmitted drug resistance* is currently increasing in the region.^55^ Such drug resistance is most frequently associated with the NNRTI class, as the signature K103N mutation has little effect on viral fitness and can therefore persist in the population even in the absence of drug pressure. Transmitted drug resistance to other drug classes is unusual; therefore, first-line therapy with a DTG-based regimen is unlikely to be affected by this phenomenon.

#### Interpreting a high viral load result of a patient receiving dolutegravir

Dolutegravir has been proved to be a remarkably robust drug in InSTI-naive patients when paired with at least one active NRTI. To date, less than five cases of DTG resistance have been described in this scenario. Thus, although a high VL has traditionally been a marker of possible resistance, this paradigm no longer applies for the most part in patients receiving a DTG-based regimen, provided that:

1. The patient has not had previous exposure to InSTIs as part of a failing regimen.
2. The patient is known to have at least one fully active NRTI as part of their regimen. (Note that patients who contract HIV whilst on pre-exposure prophylaxis [PrEP] are at risk of not having a fully active NRTI backbone).
3. The patient was not recently exposed to a scenario where a drug–drug interaction would have substantially decreased DTG concentrations (e.g. RIF-based TB therapy without increasing DTG dosing frequency to 12 hourly).

Provided that none of the above conditions are met, a detectable VL should not be assumed to reflect possible resistance. Rather, it can be assumed that the detectable VL, if not fulfilling criteria for a viral blip, merely represents poor adherence, and efforts to address this should be undertaken. *We do not recommend performing resistance testing for patients on a DTG-based regimen within 2 years of commencing the drug, provided that the above conditions are met.*

## 9. Cluster of differentiation 4 cell (CD4^+^) count

### Key points

➢ All HIV-positive patients should be started on ART irrespective of their CD4^+^ counts.
➢ Cluster of differentiation 4 counts should be used only to establish whether CTX prophylaxis and sCrAg testing are required (CD4^+^ < 200 cells/µL).
➢ Monitoring ART efficacy is best established using VL, not CD4^+^ count.
➢ Most patients newly initiating ART with an abnormally low CD4 count will see a rapid initial CD4^+^ count increase (75 cells/µL – 100 cells/µL), followed by a more gradual rise thereafter (50 cells/µL – 100 cells/µL per year) until a normal CD4^+^ count > 500 cells/µL is achieved.
➢ If CD4^+^ count does not rise despite viral suppression, the ART regimen does not need to be altered. This phenomenon may reflect an ‘immunological discordant response to ART’; however, if the patient is unwell, then other secondary causes should be sought.

### Role of cluster of differentiation 4 count monitoring

A CD4^+^ count < 200 cells/mL indicates the need for CTX prophylaxis, principally to prevent *Pneumocystis jirovecii* pneumonia, although CTX is also active against other opportunistic pathogens, including *Toxoplasma gondii, Cystoisospora belli* and *Nocardia* spp. A baseline CD4^+^ count < 200 cells/mL is also an indication to reflexly perform sCrAg testing. If the CD4^+^ count is > 200 cells/µL at baseline or it increases above this threshold on ART, then CD4^+^ testing can be stopped, as therapeutic monitoring on ART is best accomplished with VL, not CD4^+^ count or clinical criteria. However, if virologic or clinical failure occurs, then the CD4^+^ count should be repeated, as CTX prophylaxis should be commenced if the count drops to < 200 cells/µL on ART.

◦ **Common pitfall: Routinely checking CD4**^+^ **counts if the previous result was > 200 cells/µL.** This is unnecessary unless virological or clinical failure subsequently occurs.

### Timing of cluster of differentiation 4 count measurements

Cluster of differentiation 4 counts should be performed:

- at baseline (to guide decisions about CTX prophylaxis)
- every 6 months thereafter if the previous CD4^+^ count was < 200 cells/µL.

### Cluster of differentiation 4 count response

In patients who start ART with an abnormally low CD4 count, the CD4^+^ count typically increases rapidly in the first month of ART, by ~75 cells/µL – 100 cells/µL, with a more gradual rise thereafter (50 cells/µL per year – 100 cells/µL per year).^56^ Most patients achieve a CD4^+^ count > 500 cells/µL after several years of ART, provided that the VL remains suppressed. However, CD4^+^ count responses are highly variable and may fail to increase despite virological suppression in about 10% – 20% of patients.^57,58^ Such patients have a delayed or absent CD4^+^ count response to ART despite viral suppression, which is termed an ‘immunological discordant response to ART’, previously ‘immune non-responders’. Some studies have suggested that older patients are at a higher risk of this response. There is no evidence that such patients benefit from a change in ART regimen; therefore, the same regimen should be continued. Cotrimoxazole prophylaxis should be continued if the CD4^+^ count remains < 200 cells/µL. There is evidence that the prognosis of such patients is worse than in those who have a CD4^+^ response, but better than that of patients experiencing both virological and immunological failure.^58^ If patients with an immunological discordant response to ART are clinically unwell, then TB or lymphoma should be considered as the cause of persistent CD4^+^ lymphopenia. Cluster of differentiation 4 counts may remain stable in the presence of incomplete viral suppression in patients receiving ART until the VL is high (approximately ≥ 10 000 copies/mL).^59^

◦ **Common pitfall: Confusing an ‘immunological discordant response to ART’ with treatment failure.** There is no role for changing ART if the VL is suppressed.

**FIGURE 2:**
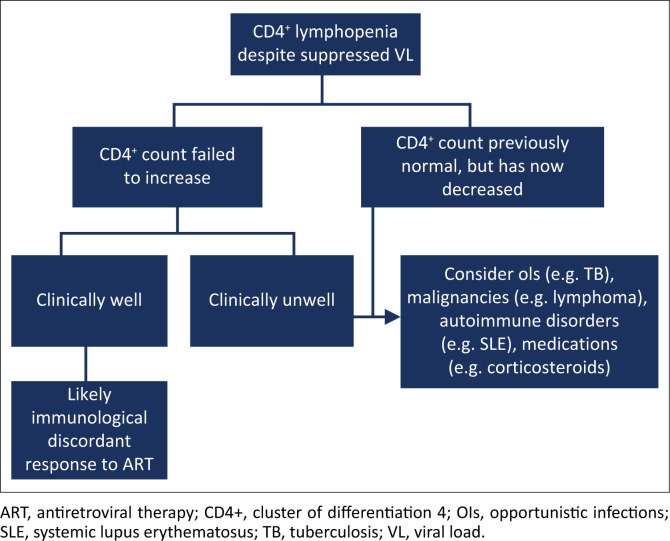
Suggested approach to patients with low cluster of differentiation 4 counts despite a suppressed viral load on antiretroviral therapy.

Figure 2 shows the outline of the suggested approach to patients with low CD4^+^ counts despite a suppressed VL on ART.

## 10. Resistance and genotyping

### Key points

➢ Adherence is the key to prevent drug resistance.
➢ Resistance testing in patients failing a DTG-based therapy is unnecessary in the majority of cases and should only be undertaken if specific criteria are met.
➢ Resistance testing may not detect archived mutations to particular drugs if the patient is not receiving these drugs at the time of resistance testing.

### Overview

As a result of transcription errors and recombination, HIV that is replicating can accumulate mutations, leading to drug resistance. Durable viral suppression by ART is required to limit the chances of developing drug resistance. Intermittent drug adherence, as opposed to a total lack of ART, provides a greater opportunity for resistance to develop, by exposing replicating virus to sub-therapeutic ART drug concentrations.

Antiretroviral drug resistance mutations are summarised in Table 9.

**TABLE 9:** Antiretroviral drug resistance mutations.

Drug	Key mutations selected
3TC or FTC	Selects for M184V, which compromises both 3TC and FTC and slightly impairs the activity of ABC but increases susceptibility to AZT and TDF.
TDF	Selects for K65R, which compromises TDF and ABC but increases susceptibility to AZT. Tenofovir disoproxil fumarate also selects for K70E, which causes low-level resistance to TDF, ABC and possibly 3TC/FTC.
ABC	Selects for L74V, which compromises ABC. May also select for K65R, which compromises TDF and ABC but increases susceptibility to AZT. Selects for Y115F, which decreases its susceptibility.
AZT	Selects for TAMs, which may ultimately compromise all NRTIs.
d4T	Selects for TAMs, which may ultimately compromise all NRTIs.
EFV or NVP	Selects for K103N, which causes high-level resistance to EFV and NVP. Also selects for Y181C and other NNRTI mutations, which cause resistance to EFV, NVP, RPV and ETR.
RPV	Selects for several mutations, including E138K, which compromise its susceptibility.
PIs	Multiple mutations are usually required before seeing a decrease in susceptibility, especially for LPV and DRV, and cross-resistance between the PIs is common. ATV selects for I50L, which causes high-level resistance to ATV but not to other PIs.
RAL	Selects for Q148H/K/R, Y143C and N155H, which cause resistance to RAL and, in certain combinations, to DTG too.
DTG	Very rarely selects resistance if InSTI-naive, provided that it is coupled with at least one other fully active drug. In patients with prior RAL exposure, mutations such as Q148H may cause decreased DTG susceptibility when combined with additional mutations.

3TC, lamivudine; ABC, abacavir; ATV, Atazanavir; AZT, zidovudine; d4T, stavudine; EFV, efavirenz; ETR, etravirine; DTG, dolutegravir; FTC, emtricitabine; InSTI, integrase strand transfer inhibitor; LPV, lopinavir; NVP, nevirapine; PIs, protease inhibitors; RAL, raltegravir; RPV, rilpivirine; TAMs, thymidine analogue mutations; NRTIS, nucleoside reverse transcriptase inhibitors; NNRTI, non-nucleoside reverse transcriptase inhibitor; TDF, tenofovir disoproxil fumarate.

### When to perform a resistance test

#### Baseline resistance test

A baseline resistance test is not generally indicated. We recommend a baseline resistance test to guide first-line regimen choice only in the following situations:

1. Pre-exposure prophylaxis received in the previous 6 months
2. History of sexual exposure to a person with known drug-resistant HIV or known to have failed an ART regimen.

#### Resistance testing at treatment failure

Resistance testing is generally only possible if the VL is > 500 copies/mL. Patients with two or more consecutive VL results of 50 copies/mL – 500 copies/mL are, however, still considered to have a virological failure (see section 8).

Recommendations for resistance testing are summarised in Table 10.

**TABLE 10:** Recommendations for resistance testing.

Regimen	DTG-based therapy	NNRTI-based therapy	PI-based therapy
First-line regimen	**Recommended** if the patient has been on regimen for > 2 years	Not routinely recommended	Not routinely recommended (rare scenario)
Second-line regimen	**Recommended** if the patient has been on regimen for > 2 years	**Recommended** (rare scenario)	**Recommended** if the patient has been on regimen for > 2 years

DTG, dolutegravir; NNRTI, non-nucleoside reverse transcriptase inhibitor; PI, protease inhibitor.

#### First-line therapy

**Non-nucleoside reverse transcriptase inhibitor-based therapy:** A resistance test at failure of first-line therapy is not routinely recommended. The EARNEST and SELECT trials showed that without the use of a resistance test to decide which NRTIs to use in second-line therapy, virological outcomes were good and equivalent to a boosted PI + RAL regimen.^60,61^ However, where funds permit, resistance testing will offer some advantages:

- A resistance test that shows no drug resistance may prevent having to switch unnecessarily to a second-line therapy.
- A resistance test may permit recycling of some first-line NRTIs (e.g. TDF), if they are shown to be susceptible. This is particularly useful if one wants to carry through TDF (or ABC) to a second-line DTG-based regimen.
- A resistance test will identify drug resistance that may be important to identify should the patient require a third-line ART in future.

**Dolutegravir-based therapy:** Because resistance to DTG in first-line therapy is extremely uncommon, we do not recommend resistance testing unless the patient has been on a first-line DTG-based regimen for more than 2 years, provided that they were not exposed to a scenario where a drug–drug interaction would have substantially decreased DTG concentrations (e.g. RIF-based TB therapy without increasing DTG dosing frequency to 12 hourly).^62^ (Other rare indications for performing a resistance test before 2 years include patients who were infected with HIV whilst receiving PrEP – see section 11). *Because of the extreme rarity of first-line DTG-based resistance mutations, we suggest switching from a DTG-based first-line regimen to a second-line regimen only if a resistance testing shows DTG resistance.*

#### Second-line therapy

##### Non-dolutegravir-containing regimens

- Resistance testing is recommended upon failure of a second-line therapy. This enables clinicians to individualise a treatment regimen for a third-line ART.
- For PI-based regimens, sufficient resistance mutations to cause virological failure typically take at least 2 years to develop; therefore, in most cases, we recommend only performing a resistance test after the patient has been on a PI-based regimen for at least this duration. Exceptions include exposure to sub-therapeutic PI drug levels as a result of drug–drug interactions (e.g. not doubling the dose of LPV/r when using RIF-based TB treatment). Patients on PI-based therapy with a VL of 50 copies/mL – 500 copies/mL pose a challenge as resistance testing is generally not possible. Such patients should remain on the same regimen with 2–3 monthly VL testing. If the VL rises to > 500 copies/mL, then resistance testing should be performed, whereas if the VL re-suppresses to < 50 copies/mL, then the patient may revert to 6–12 monthly VL testing.

**Dolutegravir-based therapy:** We do not recommend performing resistance testing for DTG-based second-line therapies within 2 years where at least one active NRTI is present. For instance, if the patient’s first-line NRTIs were FTC and TDF, and the patient was changed to 3TC and AZT, then the strain of HIV can be assumed to be fully susceptible to AZT.

Scenarios in which to consider resistance testing when failing on a DTG-containing regimen include the following:

- The patient previously developed resistance to other InSTIs (e.g. RAL).
- The ART regimen may not contain any fully active NRTIs.
- Accidental exposure to sub-therapeutic levels of DTG (e.g. RIF-based therapy was commenced without the DTG being given twice daily).

### Guide for interpreting a resistance test

Current commercial tests have been licensed for specimens with a VL of at least 1000 ribonucleic acid (RNA) copies/mL. Nevertheless, many in-house assays can detect VLs of 500 RNA copies/mL – 1000 RNA copies/mL. In general, most commercial HIV resistance tests detect mutations if they are present in > 10% – 20% of the HIV subpopulations in the sample.

◦ **Common pitfall: Performing a resistance test in patients with a low or undetectable VL.** Commercial assays may not be successful in samples where the VL is < 500 copies/mL – 1000 copies/mL.

A key concept in interpreting resistance tests is *archived resistance*. After reverse transcription from its RNA template, HIV inserts a DNA copy of itself into the host genome. Some of the cells that HIV infects are extremely long-lived, and essentially provide an ‘archive’ of HIV variants over time. *Thus, mutations that are known to have been present at one point in time can be assumed to be present for the lifetime of the patient, even if they are not visible on the patient’s latest resistance test.*

A second key concept is that of the *wild-type virus*, which is the naturally occurring HIV strain free of drug resistance mutations. In most cases, this form of the virus replicates more efficiently than viral strains that have acquired resistance. Therefore, when drug pressure is removed, the wild-type forms of the virus will predominate, even though the resistant strains have been archived and can become predominant again later if the drug pressure subsequently changes in ways favourable to these strains.

- A prominent exception to this is the signature mutation of EFV and NVP, namely K103N, which imposes no significant fitness cost on the virus. Even after these drugs are stopped, the K103N strains may persist at detectable levels for several years.
- Resistance testing should therefore only be performed when the patient is still taking his or her ART regimen, or up to a maximum of 4 weeks after discontinuation (see worked examples in Box 1).
- The absence of any identified resistance mutations implies that non-adherence is the cause of a raised VL.
- This does not exclude the possibility of archived resistance, however, which may only become detectable once the patient is back on ART that suppresses the wild-type strain.
- Any significant drug resistance mutations identified by resistance testing can be assumed to be present for the lifetime of the patient, even if subsequent resistance testings fail to show these mutations (as a result of worsened adherence or an ART switch, for instance).
- Conversely, it is only possible to identify mutations reliably for drugs that the patient was currently taking when the resistance testing was performed, and for drugs affected by cross-resistance. ‘Susceptible’ results to drugs for which there is no drug pressure may be unreliable because of archived resistance.
  ◦ **Common pitfall: Performing a resistance test in the absence of drug pressure.** If the patient has defaulted therapy for more than a few weeks, there is little purpose for a resistance test. In this scenario, it is highly likely that the replication of wild-type virus will overtake and obscure any resistant strain, rendering them undetectable by commercial resistance testing.

BOX 1 Worked example of resistance testing.

A patient was prescribed 3TC + TDF + EFV. When the patient failed this regimen after 1 year, the regimen was switched to 3TC + AZT + LPV/r. After 2 years, the patient failed this regimen too; therefore, resistance testing was performed. The results showed the following:

**Table:** 

3TC/FTC:	Resistant
TDF:	Susceptible
ABC:	Low-level resistance
AZT:	Resistant
EFV:	Resistant
LPV/r:	Low-level resistance

Interpretation:

- Since the patient was receiving 3TC + AZT + LPV/r at the time of resistance testing, it is possible to interpret the results reliably for these drugs. All three drugs show at least a low level of resistance; thus, the patient should be switched promptly to an alternative regimen.
- Efavirenz shows resistance despite the patient not receiving the drug at the time of testing. This phenomenon is not uncommon with the K103N mutation.
- Tenofovir disoproxil fumarate shows susceptibility. The patient was previously exposed to TDF, however. Although it is possible that none of the patient’s HIV strains have evolved TDF resistance, the patient was not receiving the drug at the time of resistance testing. Consequently, the possibility of archived resistance to TDF cannot be excluded.

3TC, lamivudine; TDF, tenofovir disoproxil fumarate; EFV, efavirenz; AZT, zidovudine; LPV/r, lopinavir/ritonavir; ABC, abacavir; HIV, human immunodeficiency virus.

## 11. Initial antiretroviral therapy regimens for the previously untreated patient

### Key points

➢ In ART-naive patients, the preferred initial regimen is TDF (300 mg) + 3TC (300 mg) (or FTC 200 mg) + DTG (50 mg) daily – available as a once-daily, one-tablet FDC.
➢ In patients receiving RIF, DTG dosing needs to be increased to 50 mg twice-daily until 2 weeks after stopping RIF.
➢ Dolutegravir has been associated with a small excess risk of NTDs in women taking the drug during conception – WOCP should be counselled accordingly.

### Preferred initial antiretroviral therapy regimen

The preferred initial regimen for previously untreated patients is summarised in Table 11.

**TABLE 11:** Preferred initial antiretroviral therapy regimen for previously untreated patients.

First drug	Second drug	Third drug
TDF 300 mg daily	3TC 300 mg daily, or FTC 200 mg daily	DTG 50 mg daily

3TC, lamivudine; DTG, dolutegravir; FTC, emtricitabine; TDF, tenofovir disoproxil fumarate.

In patients receiving RIF, the DTG dose needs to be increased to 50 mg twice daily until 2 weeks after stopping RIF. Other drug–drug interactions with DTG are discussed in section 3 and section 17.

Reasons for this preferred regimen are:

- This combination is available as a once-daily, one-tablet FDC from several suppliers.
- Tenofovir disoproxil fumarate is preferred over ABC because of the risk of hypersensitivity reactions with ABC (HLA-B*5701 testing is not widely available in South Africa), certain studies showing lower VL suppression with ABC when baseline VL is > 100 000 copies/mL (although not confirmed in a meta-analysis)^6^ and cost.
- Lamivudine and FTC are regarded as interchangeable in terms of efficacy and safety.
- Dolutegravir is preferred over RAL and RPV because of its higher resistance barrier.^63^ Raltegravir also requires a twice-daily dosing and is not co-formulated in FDC. Dolutegravir is preferred over EFV and PIs because superior efficacy and tolerability were demonstrated in clinical trials.^12,13^
- There is an increasing prevalence of pre-treatment resistance to NNRTIs in South Africa (> 10% in some studies), which may compromise the efficacy of EFV-based regimens.^64^

Dolutegravir has been associated with a small but significant risk of NTDs in women taking the drug during conception (0.3% vs. 0.1% in women on EFV at conception in a large Botswana birth outcomes surveillance study).^18^ This risk is lower than what was originally reported (see section 19). Women of childbearing potential should be counselled about the risks and benefits of DTG and allowed to make an informed decision regarding the use of DTG and contraception, in line with WHO 2019 recommendations. Dolutegravir has also been associated with a greater weight gain than EFV.^21^ These issues have been discussed in section 3.

### Alternative initial antiretroviral therapy regimens

Alternative regimens for previously untreated patients are summarised in Table 12.

**TABLE 12:** Alternative initial antiretroviral therapy regimens for previously untreated patients.

Regimen	Notes
TDF + 3TC (or FTC) + EFV	EFV can be used at 600 mg nocte or 400 mg nocte. EFV 400 mg dose is associated with fewer side effects and less LTFU.^22^ EFV 400 mg dose is not available in FDC in South Africa. There are insufficient data to recommend the EFV 400 mg dose in patients who are pregnant and patients receiving RIF although small-cohort studies have suggested that adequate concentrations are achieved in these patients.^65,66^
TDF + 3TC (or FTC) + RPV	RPV cannot be used in patients receiving RIF. RPV should not be used in initial therapy when baseline VL is > 100 000 copies/mL.
ABC + 3TC + DTG	International guidelines recommend HLA-B*5701 testing before prescribing ABC because a negative result rules out the risk of hypersensitivity reaction. However, this genotype is very rare in people of African descent and is thus probably not indicated. In patients of non-African descent, HLA-B*5701 testing should be considered if ABC is to be used, although access to this test is limited in South Africa.

3TC, lamivudine; ABC, abacavir; DTG, dolutegravir; EFV, efavirenz; FTC, emtricitabine; LTFU, loss to follow-up; RIF, rifampicin; RPV, rilpivirine; TDF, tenofovir; VL, viral load; FDC, fixed-dose combination.

#### Alternative initial antiretroviral therapy regimens in specific clinical situations

There are specific clinical situations in which the preferred combination of TDF + 3TC (or FTC) + DTG cannot be used and the alternatives listed in Table 13 are advised instead.

**TABLE 13:** Recommended alternative initial antiretroviral therapy regimens in specific clinical situations where TDF + 3TC (or FTC) + DTG cannot be used.

Scenario	Alternative regimen
Renal impairment at baseline (CrCl < 50 mL/min)	ABC + 3TC + DTG†
Renal impairment develops on TDF	ABC + 3TC + DTG
Patient is intolerant of DTG side effects	TDF + 3TC (or FTC) + EFV (or RPV)
Pure red cell aplasia develops because of 3TC/FTC	TDF + DTG (can then add AZT when Hb has recovered) (or RPV + DTG, provided that VL is suppressed)

3TC, lamivudine; ABC, abacavir; AZT, zidovudine; CrCl, creatinine clearance rate; DTG, dolutegravir; EFV, efavirenz; FTC, emtricitabine; Hb, haemoglobin; RPV, rilpivirine; TDF, tenofovir; VL, viral load.

† , In such patients, if renal function subsequently improves (CrCl > 50 mL/min), then they can be switched to TDF + 3TC + DTG.

If both TDF and ABC are contraindicated *and* Hb is > 8 g/dL, then AZT can be considered as an alternative NRTI.

#### Considerations for two-drug first-line regimen of dolutegravir + lamivudine

This regimen was shown to have an efficacy not inferior to a three-drug regimen in RCTs.^67^ However, these trials did not include patients with a VL > 500 000 copies/mL, and there are no follow-up data beyond 3 years for this regimen. Furthermore, virological suppression was lower in patients with a CD4^+^ count ≤ 200 cells/µL. Therefore, we do not routinely recommend this regimen unless neither TDF nor ABC can be used. Importantly, hepatitis B must be excluded before considering this regimen as patients with hepatitis B must receive TDF + 3TC (or FTC) to prevent rapid emergence of 3TC resistance. The regimen should also not be used in patients receiving RIF.

## 12. Management of patients currently receiving first-line therapy

### Key points

➢ Clinicians can consider switching patients who are virologically suppressed on NNRTI-based first-line therapy from an NNRTI to DTG, whilst maintaining the same two-drug NRTI backbone.
➢ In patients who are not virologically suppressed on an NNRTI-based regimen (VL > 50 copies/mL), we do not recommend an immediate switch to DTG, but rather an enhanced adherence counselling with repeat VL measurement in 2–3 months. If the VL remains > 50 copies/mL, then these patients should be switched to a second-line DTG regimen, which includes switching the NRTI back-bone.
➢ The typical criteria of two VL measurements greater than a certain threshold are not appropriate for DTG-based regimens, despite an adherence intervention to define virological failure. Rather, in patients started on a first-line DTG regimen, we recommend switching to a second-line therapy only if there is demonstrated InSTI resistance.

### Patients currently on an efavirenz-, rilpivirine- or nevirapine-based first-line regimen

Patients on these treatment regimens should have VL measurements performed 6–12 monthly (see the sections ‘Viral load’ and ‘Laboratory monitoring of the efficacy and safety of antiretroviral therapy’). Given that DTG is now readily available, clinicians can consider switching patients who are known to have virological suppression (VL < 50 copies/mL within last 6 months) from EFV (or NVP or RPV) to DTG whilst maintaining the same two-drug NRTI backbone. If the patient is tolerating the EFV (or RPV or NVP) regimen with no side effects, then such a switch is optional, as the patient may develop DTG-related side effects which he or she was not experiencing on the NNRTI (e.g. insomnia and weight gain). The benefit of such a switch is that a DTG regimen has a more robust resistance profile (DTG boasts a higher barrier to resistance than NNRTIs).^12^ An additional benefit of switching from NVP to DTG is that it is switching from a twice-daily to a once-daily regimen. In patients experiencing EFV-related side effects (even mild side effects), we encourage a change to DTG whilst maintaining the same NRTIs, provided that the VL is < 50 copies/mL within the last 6 months.

Another option in patients who are virologically suppressed (VL < 50 copies/mL) whilst receiving a regimen of NNRTI + two NRTIs, and who have never experienced virological failure, is a switch to the two-drug combination of DTG + RPV. Data from two clinical trials (SWORD I and II)^68^ showed that this regimen maintains virological suppression as a switch strategy in patients who have not previously experienced virological failure. This should not be done in patients who have chronic hepatitis B as TDF and 3TC (or FTC) should always form a part of their treatment.

**FIGURE 3:**
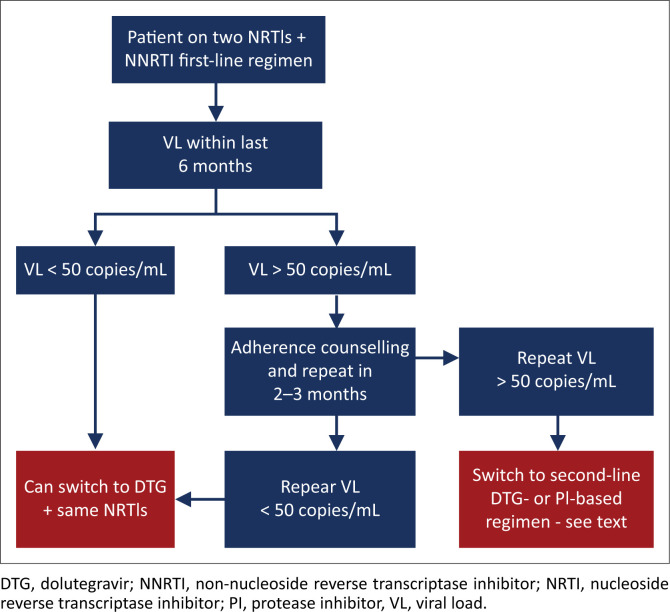
Switching from a first-line non-nucleoside reverse transcriptase inhibitor-based regimen to a dolutegravir-based regimen.

The recommended protocol for switching from a first-line NNRTI-based regimen to a DTG-based regimen is shown in Figure 3.

#### Antiretroviral therapy options in patients failing first-line non-nucleoside reverse transcriptase inhibitor-based therapy

In patients who are not virologically suppressed on an NNRTI-based regimen (VL > 50 copies/mL), we do not suggest an immediate switch to DTG with the same two NRTIs. The reason for this is that it is possible that they have developed resistance to the two NRTIs and may then be placed on DTG without a fully effective drug to accompany it. In this scenario, we recommend for enhanced adherence counselling and repeating the VL measurement in 2–3 months. If the VL is < 50 copies/mL, then the patient can be switched to DTG + the same two NRTIs. If the VL is > 50 copies/mL, then the patient should be switched to a second-line DTG regimen, which includes DTG + two NRTIs as follows:

- If the patient was receiving TDF (or ABC) + 3TC (or FTC) first-line therapy, then switch to AZT + 3TC in a second-line therapy with DTG.
- If the patient was receiving AZT (or D4T) + 3TC first-line therapy, then the decision regarding a second-line therapy should be based on a resistance test result. If the virus is susceptible to TDF on resistance testing, then the clinician can prescribe TDF + 3TC (or FTC) + DTG (provided that the patient has not potentially previously experienced virological failure on TDF and has not experienced TDF nephrotoxicity previously). If no fully active NRTI is available to accompany DTG, then it is best to switch to a PI-based second-line therapy with TDF + 3TC (or FTC). Advice is provided in section 13 regarding patients who cannot access a resistance test in this scenario.

Women of childbearing potential should be counselled about the potential risks and benefits of DTG and allowed to make an informed decision regarding the use of DTG and contraception (see section 3).

### Patients started on a dolutegravir-based first-line regimen

These patients should have their VL measured 6–12 monthly (see the sections ‘Viral load’ and ‘Laboratory monitoring of the efficacy and safety of antiretroviral therapy’). We have previously used the criteria of two VL measurements > 1000 copies/mL despite an adherence intervention to define virological failure and the need to switch from first- to second-line ART. This was appropriate for patients on NNRTI-based first-line regimens because of the low barrier to resistance of the NNRTI class. However, considerations are very different with DTG-based first-line regimens. In several clinical trials of DTG in first-line therapy, no DTG resistance has been described despite some patients having virological failure, and in clinical practice very few cases of DTG resistance (less than five cases worldwide at the time of writing this article) have been described when the drug has been used as part of a three-drug first-line regimen.^63^ Therefore, it would be inappropriate to use the same criteria for switching to second-line ART for DTG as it is likely that most patients with two unsuppressed VLs will not have resistance and rather require improved adherence on the same first-line regimen to achieve suppression. For that reason, we only recommend switching from a first-line DTG-based ART to a second-line regimen if resistance testing demonstrates InSTI resistance.

Until further data are available, in patients with an unsuppressed VL on DTG-based first-line ART, we recommend for enhanced adherence counselling. The tolerance of the regimen should also be addressed – the regimen may need to be switched because of side effects. Integrase strand transfer inhibitor resistance testing should be considered in these situations:

- Dolutegravir monotherapy for a period (DTG resistance has been more frequently described in this situation).^63^
- Co-administration of a drug that interacts with DTG without necessary dose adjustment (e.g. DTG given at 50 mg daily with RIF or given simultaneously with polyvalent cation-containing agents – see section 3).
- Viral load measurements > 50 copies/mL for > 2 years (despite adherence interventions, 100% pharmacy refills and self-reported adherence) and the current VL is > 500 copies/mL, thereby permitting a resistance test.
- The patient was infected whilst receiving PrEP (because of potential NRTI resistance at the time of infection).
- Sentinel surveillance projects or research studies, with the purpose of detecting emergence of DTG resistance.

If a resistance test is performed, then this should include sequencing of the integrase enzyme. The clinician should only switch from a DTG-based first-line regimen to a second-line therapy if resistance is detected, and the drugs used in the second-line regimen should depend on the resistance test result (see section 13).

These recommendations are based on accumulated information on the resistance barrier to DTG to date, suggesting that DTG resistance is extremely rare when the drug is used in a three-drug first-line regimen. These recommendations may be updated when more data become available regarding the incidence and risk factors of DTG resistance with more widespread use in routine clinical practice.

**FIGURE 4:**
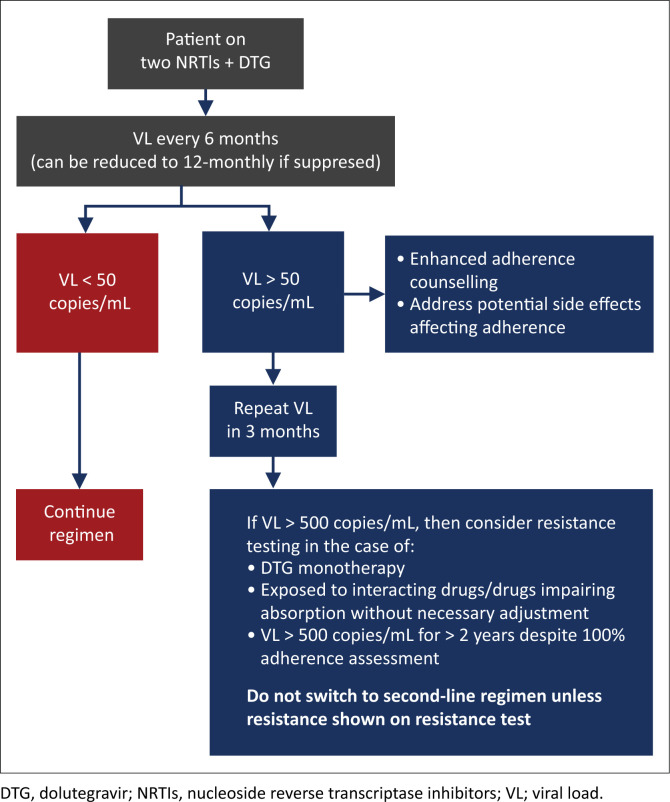
Virological monitoring of patients receiving dolutegravir-based first-line antiretroviral therapy and response to results.

Figure 4 shows the outline of the virological monitoring of patients on DTG-based first-line ART and the recommended response to results.

## 13. Management of patients starting or currently receiving second-line therapy

### Key points

➢ When DTG is used in second-line therapy, there should be at least one fully active accompanying drug until further evidence is available.
➢ If the patient fails an NNRTI regimen with 3TC/FTC + either TDF or ABC, then AZT + 3TC + DTG is the recommended second-line regimen.
➢ If the patient fails other first-line regimens, then resistance testing is advised to decide on the choice of NRTIs in a DTG-based second-line regimen.
➢ Boosted PI + two NRTI second-line regimens are effective even if there is resistance to both NRTIs in the regimen.
➢ We advise DRV/r 800 mg/100 mg once daily as the first choice PI for use in second-line therapy.

### Recommendations for patients failing a first-line regimen

#### Failed first-line regimen of two nucleoside reverse transcriptase inhibitors + non-nucleoside reverse transcriptase inhibitor

Table 14 shows the summary of the recommended second-line regimen to start in patients who have failed a first-line regimen consisting of two NRTIs + NNRTI.

**TABLE 14:** Recommended second-line regimen in patients who have failed a first-line regimen of two nucleoside reverse transcriptase inhibitors + non-nucleoside reverse transcriptase inhibitor.

Failing first-line regimen	Advised second-line regimen
TDF + 3TC (or FTC) + NNRTI	AZT + 3TC + DTG‡
AZT + 3TC + NNRTI	Resistance test: if fully active NRTI is available, then combine this with: 3TC (or FTC) + DTG, or TDF + FTC + DRV/r†
ABC + 3TC + NNRTI	AZT + 3TC + DTG‡

NNRTI, non-nucleoside reverse transcriptase inhibitor; NRTI, nucleoside reverse transcriptase inhibitor; TDF, tenofovir disoproxil fumarate; 3TC, lamivudine; AZT, zidovudine; ABC, abacavir; DTG, dolutegravir; FTC, emtricitabine; DRV, darunavir.

† , If the patient has chronic hepatitis B, then continue TDF, in addition, in the second-line regimen.

‡ , Provided that the patient has not potentially previously experienced virological failure on TDF, and has not experienced TDF nephrotoxicity previously.

Based on the results of the DAWNING trial, it is preferable to use a DTG-based regimen rather than a PI/r regimen in second-line therapy.^17^ In this trial, a second-line regimen of DTG + two NRTIs was superior in terms of virological suppression and better tolerated than LPV/r + two NRTIs in patients who had failed a first-line regimen of NNRTI + two NRTIs. An important caveat is that all patients enrolled in this trial had a resistance test performed at entry and had to have at least one fully active NRTI to be eligible for inclusion. *Thus, the current evidence supports a DTG-based regimen in second line only when used with at least one fully active NRTI.* Whether a DTG-based second-line regimen would be equally effective with two NRTIs when there is resistance to both those NRTIs is currently a knowledge gap that is being addressed by several clinical trials – we do not advise such a strategy until the results of those trials are available.

In patients failing a first-line regimen in which the NRTIs are TDF + 3TC (or FTC), the mutations selected are typically M184V by 3TC (or FTC) and K65R (or K70E) by TDF. None of these mutations compromise AZT; in fact, they render the virus hyper-susceptible to it. Therefore, in this scenario, AZT remains fully active, and we can infer from the DAWNING trial results that a second-line regimen of AZT + 3TC + DTG will be optimally effective. The same applies for patients failing an ABC + 3TC + NNRTI regimen: AZT retains susceptibility and can be used with 3TC and DTG in a second-line regimen.

Where patients have failed an AZT (or d4T) + 3TC + NNRTI regimen, this could have resulted in the accumulation of thymidine analogue mutations (TAMs) and M184V. Certain TAMs compromise TDF, meaning that it is unpredictable whether there is a fully active NRTI if a regimen of TDF + 3TC (or FTC) + DTG is used in second-line therapy. We therefore advise resistance testing in such patients. If the resistance test demonstrates a fully active NRTI, then that NRTI can be used with 3TC (or FTC) + DTG in the second-line regimen. If there is no fully active NRTI or a resistance test is not possible in this situation (e.g. public sector), then we recommend a regimen of TDF + 3TC (or FTC) + DRV/r. The reason for this is that boosted PI + two compromised NRTIs retain activity as a second-line regimen (see below).

Women of childbearing potential should be counselled about the risks and benefits of DTG and allowed to make an informed decision regarding the use of DTG and contraception. If they choose not to use DTG in a second-line therapy, then DRV/r should be used in its place.

#### Failed first-line regimen of two nucleoside reverse transcriptase inhibitors + dolutegravir

The second-line regimen to commence in patients who have failed a first-line approach of two NRTIs + DTG is provided in Table 15.

**TABLE 15:** Recommended second-line regimen in patients who have failed a first-line regimen of two nucleoside reverse transcriptase inhibitors + dolutegravir.

Failing first-line regimen	Advised second-line regimen
Two NRTIs + DTG	Only switch to second-line regimen if the resistance test shows DTG resistance and the second line should be two NRTIs (selected based on resistance test) + DRV/r

DTG, dolutegravir; NRTI, nucleoside reverse transcriptase inhibitor; DRV, darunavir/ritonavir.

If patients experience virological failure on a first-line DTG-based regimen, then we do not recommend switching to a second-line therapy unless a resistance test is performed that demonstrates DTG resistance. This is because DTG is a very robust drug and resistance is very rare when used in triple-drug combination first-line therapy. Therefore, it is far more likely that a VL > 50 copies/mL is attributed to adherence problems rather than resistance. If DTG resistance is demonstrated, then we advise a regimen of two NRTIs + DRV/r, with the two NRTIs selected based on the resistance test results.

### Patients currently established on protease inhibitor-based second-line therapy

#### Viral load < 50 copies/mL

Clinicians can consider switching of patients currently on a second-line PI/r regimen to a DTG regimen, particularly if patients experience GI side effects of PIs. This switch may also simplify the regimen and reduce pill burden. However, before such a switch is made, we advise a careful review of the treatment and resistance (genotype test) history to ensure that there is at least one fully active NRTI to accompany DTG in the new regimen. If this cannot be assured, then we advise maintaining the current PI/r regimen – although a switch to an alternative PI/r can be considered to improve tolerance. If continuing a PI is not possible, then a switch to a DTG-based second-line regimen without an assured active NRTI could be considered with close VL monitoring. Refer to Table 16 for our advice when considering switching a patient on a PI/r-based regimen to DTG in a second-line therapy where the VL is < 50 copies/mL.

**TABLE 16:** Switching from a boosted protease inhibitor to dolutegravir in second-line antiretroviral therapy when the viral load is < 50 copies/mL.†

First- and second-line regimen: Prior antiretroviral therapy exposure	Second-line options
First-line TDF + 3TC (or FTC) + NNRTI and second-line AZT + 3TC + PI/r	Can continue the same regimen or switch to AZT + 3TC + DTG.
First-line AZT (or d4T) + 3TC + NNRTI and second-line TDF + FTC + PI/r	Preferably stay on the same regimen. If resistance testing was performed at first-line failure and showed full susceptibility to TDF, then can switch to TDF + 3TC (or FTC) + DTG. If no resistance test was performed, but there is intolerance to all boosted PIs, then consider switching to TDF + 3TC (or FTC) + DTG with close virological monitoring (3 monthly) for the first year.

Note: See Box 2 and 3 for more information. TDF, tenofovir disoproxil fumarate; 3TC, lamivudine; FTC, emtricitabine; NNRTI, non-nucleoside reverse transcriptase inhibitor; AZT, zidovudine; PI/r, ritonavir-boosted protease inhibitors; DTG, dolutegravir.

† , ABC is interchangeable with TDF in this table.

BOX 2 Choice of boosted protease inhibitor in second-line antiretroviral therapy.

If a boosted PI is used in second-line therapy, then this is our recommendation in the order of preference:

1. DRV/r 800 mg/100 mg daily
2. ATV/r 300 mg/100 mg daily
3. LPV/r 400 mg/100 mg twice daily

The first choice PI is DRV/r 800 mg/100 mg daily. The once-daily rather than twice-daily dosing can be used in patients who are PI-naive. The once-daily dosing may facilitate adherence and be better tolerated.^69^

Darunavir is better tolerated than LPV/r. It has a similar risk of GI intolerance and lipid abnormalities when compared with ATV/r, but does not cause hyperbilirubinaemia like ATV. Darunavir/ritonavir also has a high genetic barrier to resistance.

Darunavir/ritonavir 400 mg/100 mg daily has also been evaluated in switch studies for patients who are suppressed on a PI/r and who have not previously failed a PI. This lower dose of DRV/r retained virological suppression in the vast majority of patients in two trials^33,34^ and in a similar proportion to those who continued LPV/r in one of these trials.^33^ Clinicians could consider switching second-line patients who are suppressed on a PI/r to this dose, particularly if cost is an issue.

Darunavir/ritonavir cannot be co-prescribed with RIF and the alternatives to this in second-line therapy are DTG 50 mg twice daily (see above regarding one fully active NRTI), double-dose LPV/r or switching RIF to rifabutin (see section 18).

Based on clinical trials demonstrating superior tolerability, if DRV/r cannot be used, then we suggest that ATV/r 300 mg/100 mg daily is preferred over LPV/r.^70,71^ The benefits of ATV/r over LPV/r include improved tolerance in terms of GI side effects, a more favourable lipid profile and once-daily administration. Drawbacks of ATV/r are the following: it cannot be used with RIF-based TB treatment, and there are important drug interactions with drugs that reduce stomach acidity such as PPIs. An alternative PI/r rather than ATV/r should be used in the following situations:

Patients who do not tolerate ATV/r (e.g. cosmetically unacceptable jaundice).

Patients receiving RIF-based TB treatment: the alternatives to this in second-line therapy are DTG 50 mg twice daily (see above regarding one fully active NRTI), double-dose LPV/r or switching RIF to rifabutin (see section 18).

Lopinavir is the third choice PI/r. It is co-formulated with RTV in a heat-stable tablet (Aluvia). Lopinavir/ritonavir is an option with RIF when double-dosed. Darunavir/ritonavir and ATV/r can never be co-administered with RIF-based TB treatment.

DRV/r, darunavir/ritonavir; ATV/r, atazanavir/ritonavir; GI, gastrointestinal; LPV/r, lopinavir/ritonavir; PI, protease inhibitor; PI/r, ritonavir-boosted protease inhibitors; DTG, dolutegravir; NRTI, nucleoside reverse transcriptase inhibitor; RIF, rifampicin; TB, tuberculosis; PPI, proton pump inhibitor; RTV, ritonavir.

BOX 3 Boosted protease inhibitor + two nucleoside reverse transcriptase inhibitors is an option in second-line therapy even if there is resistance to both nucleoside reverse transcriptase inhibitors.

Because boosted PIs are robust drugs (i.e. resistance develops slowly) in PI-naive patients, it is very likely that virological suppression will be achieved with good adherence, even if the two nucleoside reverse transcriptase inhibitors (NRTIs) used in second-line therapy are compromised by NRTI resistance mutations. This is supported by the findings of the Europe-Africa Research Network for Evaluation of Second-line Therapy (EARNEST)^60^, Second-Line Effective Combination Therapy (SELECT) (in resource-limited settings)^61^ and SECOND-LINE^72^ trials, which showed good virological suppression rates of second-line LPV/r and NRTI regimens, even in patients with significant NRTI resistance. These trials demonstrated that without the use of a resistance test to decide which NRTIs to use in second-line therapy with ritonavir-boosted ritonavir (PI/r), virological outcomes were good and similar to a boosted PI + RAL regimen. In addition, those patients with more extensive NRTI resistance at first-line failure were more likely to achieve virological suppression in second-line therapy.^73^

PIs, protease inhibitors; RAL, raltegravir; NRTI, nucleoside reverse transcriptase inhibitor; LPV/r, lopinavir/ritonavir.

#### Viral load > 50 copies/mL

In patients on a second-line regimen containing a boosted PI, if the VL is not suppressed, then we do not advise switching to DTG. We advise for enhanced adherence counselling and switching to an alternative boosted PI if there is intolerance. If the VL subsequently re-suppresses to an undetectable VL < 50 copies/mL, then the advice in the section above should be followed. If the VL remains elevated, then the patient may be eligible for a resistance test, with consideration for a third-line therapy if they fulfil the criteria outlined in section 14.

## 14. Third-line antiretroviral therapy

### Key points

➢ For patients with a detectable VL on second-line therapy for < 2 years, intensified adherence counselling and support are required rather than a switch to a third-line therapy.
➢ For a patient on a second-line regimen for > 2 years with two or three VL measurements > 50 copies/mL within a 6-month period despite adherence interventions that have been assessed to be satisfactory (see text), a resistance test should be performed. (N.B.A resistance test can only be performed if VL > 500 copies/mL.).
➢ The choice of the third-line regimen should be made in consultation with an HIV expert.

### Management of patients with a detectable viral load on second-line therapy and initiation of third-line therapy

As outlined in the previous section, there should be documented PI or DTG resistance before switching to a third-line regimen. Resistance tests should be interpreted by an expert in conjunction with a full ART history. In many patients failing second-line regimens, there are no PI (or DTG) mutations. In these patients, improved adherence is required rather than switching to a third-line regimen. If side effects interfere with adherence, then consideration should be given to switching to a more tolerable regimen, provided that this regimen is predicted to be effective based on the treatment history (see section 13).

**FIGURE 5:**
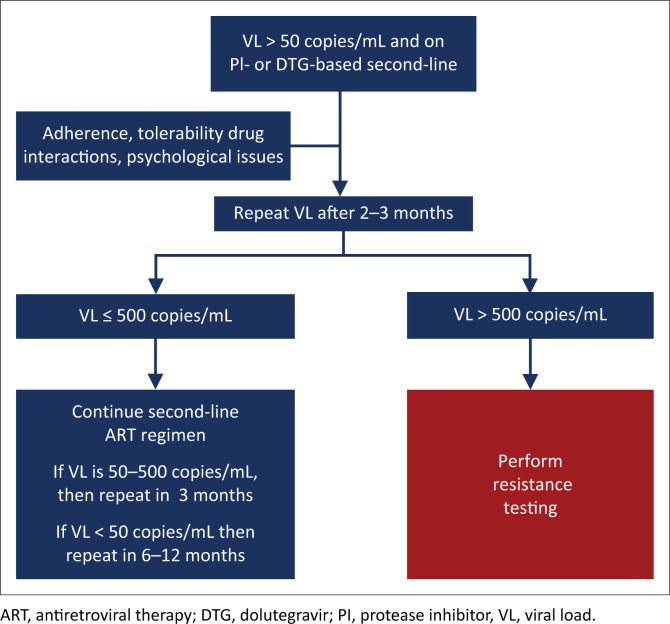
Indications for performing resistance (genotype) testing in second-line antiretroviral therapy.

Figure 5 shows the outline of indications for performing resistance testing in second-line ART regimens.

In a patient who has been on a second-line regimen for > 2 years, if there are two or three VL measurements > 50 copies/mL in a 6-month period, despite adherence interventions, and adherence is assessed to be satisfactory (e.g. 100% pharmacy claims over 6 months), then a resistance test should be performed (resistance test can only be performed if VL > 500 copies/mL). If a patient who has been on second-line therapy for < 2 years is found to have a detectable VL, then a resistance test should not be performed; rather, the same regimen should be continued and adherence counselling and support should be intensified. The regimen may be switched if there are significant side effects. It is unlikely that significant resistance to the PI or DTG will have developed within 2 years. The exceptions include a patient who in error was not prescribed LPV/r double dosing with concurrent RIF use, and subsequently demonstrates a detectable VL, and a patient who has been taking an incorrectly low dose of medication. Such patients should be eligible for resistance testing even if they have been on second-line therapy for < 2 years.

### Adherence counselling before third-line therapy

Specific adherence counselling should be provided for patients preparing to start third-line ART, with a clear discussion that this regimen is likely to be their last option for the foreseeable future.

### Third-line regimen choice after failing a protease inhibitor-based second-line regimen

A third-line regimen including a combination of DRV/r and other drugs decided on the basis of resistance testing results in virological suppression in the majority of patients, provided that adherence is optimal.^74,75,76,77^

For most patients who require third-line therapy (i.e. patients who experience virological failure on a second-line LPV/r or ATV/r regimen with a low-, intermediate- or high-level resistance to the PI, i.e. Stanford Score > 14), we recommend the third-line regimen outlined in Table 17. However, the final decision will be based on treatment history and resistance test results for the individual patient.

**TABLE 17:** Third-line regimen recommended for the majority of patients failing protease inhibitor-based second-line therapy.

Failing second-line regimen	Advised third-line regimen
Protease inhibitor-based therapy	TDF 300 mg daily, 3TC 300 mg daily, DTG 50 mg daily (given as TLD) **plus** DRV/r 600 mg/100 mg twice daily

3TC, lamivudine; DTG, dolutegravir; DRV/r, ritonavir-boosted darunavir; TDF, tenofovir disoproxil fumarate; TLD, tenofovir disoproxil fumarate + lamivudine + dolutegravir fixed-dose combination.

#### Exceptions

There are exceptions:

- In patients with *renal impairment*, replace TDF + FTC with ABC + 3TC, or AZT + 3TC. The choice between ABC and AZT will depend on Hb (*anaemia: do not use AZT if Hb < 8 g/dL*) and resistance testing.
- If the AZT Stanford score is lower than the TDF score, then use AZT + 3TC rather than TDF + FTC.
- Patients with a DRV score of 0 on Stanford score (and no DRV mutations, see section 5) can take DRV/r 800 mg/100 mg once daily.
- Patients with prior virological failure on RAL and/or with a DTG score > 0 on integrase resistance testing should receive DTG 50 mg twice daily.
- In patients with extensive resistance (e.g. DRV score > 29 and NRTI score > 29), consider adding RPV or ETR (provided that the score for these drugs is < 30) or maraviroc (MVC) (provided that the virus is CCR5-tropic on the tropism test).

#### Additional points and explanatory notes

- We generally advise the continuation of NRTIs in the third-line regimen, even if there is documented NRTI resistance.
- Lamivudine (or FTC) resistance with the M184V mutation impairs viral replication. Another NRTI (generally TDF, but based on resistance testing) should be added. This is not essential if there are more than two other active drugs in the regimen.^78^
- Because most patients are not receiving an NNRTI at the time of failing second-line therapy when a genotype resistance test is typically performed, prior NNRTI mutations related to first-line NNRTI failure may be archived at this time. Therefore, it is difficult to be certain from a (this) genotype performed at second-line ART failure whether ETR/RPV is still active; however, data from South Africa suggest that the majority of patients who have failed NVP or EFV are still susceptible to ETR/RPV.^79^
- In the SAILING trial, in treatment-experienced patients, a DTG regimen proved superior to RAL and fewer patients in the DTG arm developed treatment-emergent InSTI resistance. *Consequently, we no longer recommend the use of RAL in third-line therapy unless DTG is not tolerated or otherwise contraindicated or unavailable*. We also recommend switching patients currently using RAL in third-line therapy to DTG because of its higher barrier to resistance.^14^ If such patients have a suppressed VL, then they can be switched to standard dose DTG (50 mg daily); however, if they are not virologically suppressed, then we suggest a resistance test with a request for integrase sequencing before switching. If there are InSTI mutations present that are associated with reduced susceptibility to DTG, then the DTG dose should be 50 mg twice daily.
- Women of childbearing potential should be counselled about the risk and benefits of DTG and allowed to make an informed decision regarding the use of DTG and contraception.
- Maraviroc (a CCR5 blocker) is a consideration in third-line therapy; however, it is currently extremely costly and can only be used after a tropism test demonstrates that the patient’s circulating virus has sole tropism for the CCR5 co-receptor. We advise only considering this when there is intermediate- or high-level resistance to all PIs, all NNRTIs and all NRTIs, and DTG is not fully susceptible.
- If viral suppression is not achieved on third-line therapy, then there is still benefit in continuing failing ART because of the residual partial activity and ‘crippling’ effect of such ART. ‘Crippling’ describes the fact that mutant viruses often have less replicative capacity. Provided that the VL can be maintained at < 10 000 copies/mL, the CD4^+^ count will usually be maintained or even increase.^59^

### Third-line regimen choice after failing a dolutegravir-based second-line regimen

The choice of a regimen will be guided by resistance test results and should include DRV/r with two NRTIs (generally 3TC or FTC with the NRTI with the lowest Stanford score). In patients failing a second-line DTG regimen who have not failed a PI/r previously, it can be assumed that DRV/r is fully active and can be used at 800 mg/100 mg daily. Based on the results of the EARNEST, SELECT and SECOND LINE trials, it can be concluded that a DRV/r regimen with two NRTIs will be an active regimen even if there is documented resistance to the two NRTIs.^60,61,72^ However, decisions regarding the third-line therapy need to be individualised in consultation with an expert, taking into account the treatment history (which drugs and classes the patient previously failed) and previous and current resistance test results.

Raltegravir may still be active in patients with DTG resistance – it depends on the specific resistance mutations in the integrase gene – but it is usually not necessary to include it in the regimen. Etravirine and RPV are other drugs that can be considered depending on resistance test results and treatment history.

## 15. Laboratory monitoring of the efficacy and safety of antiretroviral therapy

### Key points

➢ Antiretroviral therapy efficacy is monitored with VL and CD4^+^ count – discussed in the sections ‘Viral load’ and ‘CD4^+^ cell count’.
➢ The key efficacy endpoint in ART is sustained virological suppression with a VL < 50 copies/mL.
➢ CD4^+^ count monitoring can be stopped when the CD4^+^ count is > 200 cells/µL and the VL is suppressed.
➢ Creatinine monitoring is advised in patients on TDF, and an FBC is advised in patients on AZT.
➢ In most patients taking PIs, only one lipid measurement is advised (at 3 months).
➢ In patients on TDF who are admitted to hospital, it is important to check creatinine even if it does not fall within these monitoring guidelines. This is because intercurrent illnesses with dehydration or sepsis may be associated with a deterioration in renal function, in which TDF may act as a co-factor.

Table 18 shows the list of the laboratory investigations and their frequency advised for monitoring of ART safety.

- **Common pitfall: Monitoring of VL is not done at least annually.** This results in a delayed detection of ART failure and intervention, with resultant clinical deterioration and increased risk of transmission.

**TABLE 18:** Standard laboratory monitoring of patients after commencement of antiretroviral therapy.

Test†	When		Comments
	Baseline	Ongoing	
VL	Yes	At 3 months, 6 months and then 6 monthly	If VL undetectable for > 12 months, can reduce to 12 monthly
CD4^+^ count	Yes	6 monthly At virological/clinical failure	Can be stopped if CD4^+^ > 200 cells/µL *and* virologically suppressed
FBC + differential count	Yes	Monthly for the first 3 months, then at 6 months	For patients on AZT-containing regimens
ALT	Yes	At initiation	If baseline ALT is normal, routine monitoring of ALT is not required
CrCl	Yes	At 3 months, 6 months and then 6 monthly	Also at 1 and 2 months in high-risk patients. If symptoms of tubular wasting (e.g. muscle weakness), then check potassium and phosphate levels
TC and TG	Not routinely	After 3 months on a PI-containing regimen	If normal at 3 months, reassess only if other cardiovascular risk factors are present

ALT, alanine transaminase; AZT, zidovudine; CrCl, creatinine clearance rate; FBC, full blood count; NVP, nevirapine; TC, total cholesterol; TG, triglycerides; VL, viral load; PI, protease inhibitor; CD4^+^, cluster of differentiation 4.

† , These tests should also be performed when clinically indicated, based on the discretion of the clinician.

## 16. Patients who return after stopping antiretroviral therapy

### Key points

➢ Many patients return to care after treatment interruption when they experience clinical deterioration – screening for OIs should be performed.
➢ Viral load measurement should be performed before re-initiation and repeated 3–6 monthly.
➢ Choosing the appropriate regimen in patients who return to therapy depends on their previous regimen, their level of treatment adherence prior to disengaging with care, and their current CD4 and hospitalisation status.

It is common for patients receiving ART to interrupt their treatment for a number of reasons (e.g. treatment fatigue, denial, life event, depression, new job, relocation, etc.). Many patients return to care after an interruption, often precipitated by clinical deterioration. Patients who have clinical symptoms of an OI when returning to care should be investigated and, if appropriate, should be started on treatment for the infection before restarting ART. In particular, patients should be screened for headache and for TB symptoms when returning to care. Reasons for delaying ART re-initiation are the same as for delaying initiation in ART-naive patients (see section 6). Patients who are asymptomatic when returning to care could be re-initiated on ART the same day with appropriate counselling. A counselling plan should be implemented to ensure retention in care going forward and address reasons for disengagement.

We recommend performing a VL measurement before re-initiating ART, then 3–6 monthly thereafter. The choice of ART regimen to restart will depend on prior treatment history.

### Return after stopping a TDF + FTC (or 3TC) + NNRTI regimen

In patients returning to treatment after disengaging from a TDF + FTC (or 3TC) + NNRTI regimen:

- If the patient had adhered to treatment prior to disengaging, with a suppressed VL, and has only disengaged once or twice, then he or she could either be restarted on the same regimen or restarted on TDF + 3TC (or FTC) + DTG. If a patient restarts an NNRTI-based regimen, then switching to a second-line regimen should be considered if the VL is not < 1000 copies/mL at 3 months after restarting.
- *If the patient has a history of poor adherence with multiple episodes of disengaging, then we suggest re-initiating therapy with a second-line regimen of AZT + 3TC + DTG, AZT + 3TC + PI/r or TDF + FTC + PI/r*. We do not recommend TDF + 3TC (or FTC) + DTG in this scenario, as there may be resistance to TDF and 3TC – multiple episodes of treatment interruption, particularly beyond the first year of ART, and poor adherence can result in resistance to all drugs in the first-line regimen.
- *Hospitalisation with an AIDS-defining condition and a CD4*^+^ *count < 50 cells/µL* represents another scenario in which a patient may be restarted immediately on second-line ART when returning to care after disengaging. Such patients are considered to be at high risk of mortality if restarted on first-line therapy to which their virus may be resistant, and they require an ART regimen that is guaranteed to be effective immediately. This decision should typically be taken by the hospital-level clinician.

### Return after stopping a dolutegravir-based regimen

In patients returning to treatment after disengaging from a DTG-based regimen:

- Restart the same regimen. Assess the VL at 6 months and follow standard guidance in response to the result.

### Return after stopping a TDF + FTC (or 3TC) + PI/r regimen

In patients returning to treatment after disengaging from a second-line PI-based regimen:

- Either restart the patient on the same regimen or switch to AZT + 3TC + DTG (if the patient had failed a first-line TDF + 3TC (or FTC) + EFV regimen). Assess the VL at 6 months and follow standard guidance in response to the result.

### Return after stopping a third-line regimen

In patients returning to treatment after disengaging from a third-line regimen:

- Restart the same regimen and assess the VL at 6 months. Follow standard guidance in response to the result.
  - **Common pitfall: Performing a resistance test after an ART treatment interruption of > 4 weeks.** Such a testing is of limited value. Many resistance mutations are overtaken by the wild-type virus when ART is stopped and thus the resistance test may not accurately reflect the true resistance pattern.

## 17. Drug–drug interactions

### Key points

➢ Whenever patients start or switch antiretroviral drugs or start new concomitant medications, it is important to evaluate potential drug interactions.
➢ Many drugs and drug classes have clinically significant drug–drug interactions with ARVs.
➢ There are also important drug interactions between several ARVs.
➢ It is important to consult a regularly updated database to assess whether drugs can be co-administered and whether dose adjustment is required.
➢ Herbal medications may also have interactions with ART drugs (e.g. St John’s wort and garlic), but data on herb–drug interactions are very limited.

### Mechanisms of drug interactions

There are two main mechanisms of drug–drug interactions:

- *Pharmacodynamic interactions*: these interactions occur when one drug influences the action of another drug without altering its concentrations. Such interactions may be either beneficial, if drug effects are additive or synergistic; or harmful, if drug effects are antagonistic. Additive toxicity is also a pharmacodynamic interaction (e.g. AZT and linezolid both cause myelosuppression and should not be co-administered).
- *Pharmacokinetic interactions*: these interactions occur when a perpetrator drug alters the concentrations of a victim drug by affecting its absorption, distribution, metabolism or excretion. Inhibition is a direct chemical effect when a drug binds to the active site of drug-metabolising enzyme or drug transporter – typically only one or a few enzymes or transporters are inhibited. Inhibition is maximal when the inhibiting drug reaches steady state and wanes rapidly when the inhibiting drug is stopped. Strong inhibitors (e.g. ritonavir, clarithromycin and itraconazole) can cause significant increases in the concentrations of victim drugs, resulting in toxicity. Induction results in transcriptional activation of many genes involved in drug metabolism and transport, which takes about 2 weeks to be maximal and wanes in a similar time. Strong inducers (e.g. RIF, carbamazepine and phenytoin) can cause significant decreases in concentrations of victim drugs, resulting in reduced efficacy. Pharmacokinetic interactions are occasionally beneficial (e.g. RTV markedly increases the concentrations of other PIs). Data on herb–drug interactions are very limited – both St John’s wort and garlic are known inducers. Clinically significant pharmacokinetic interactions require dose adjustment of the victim drug or, if the interaction is severe, avoiding co-administration with the perpetrator drug.

### Overview of drug–drug interactions by antiretroviral class

- **NRTIs.** these are generally neither victims nor perpetrators of clinically significant pharmacokinetic interactions.
- **PIs.** ritonavir is a potent inhibitor of the key CYP enzyme 3A4 and the drug efflux transporter P-glycoprotein; it also induces several other drug-metabolising enzymes and drug transporters. Therefore, RTV-boosted PIs are frequent perpetrators of pharmacokinetic interactions, but can also be victims of such interactions when co-administered with strong inducers – co-administration with strong inhibitors does not add significantly to the inhibition by RTV. Atazanavir/ritonavir requires an acid pH in the stomach for absorption – it should be taken 2 h before or 1 h after antacids, and administration with PPIs is not advised.
- **NNRTIs.** these differ by individual drugs. Efavirenz is a moderate inducer. Rilpivirine can be the victim when co-administered with strong inducers. Although inhibitors increase the exposure to RPV, it is seldom necessary to adjust the dose. Etravirine induces CYP3A4 and also inhibits two CYP enzymes; it can also be the victim when co-administered with strong inducers.
- **InSTIs.** Polyvalent cations (calcium, magnesium, iron and aluminium) bind to InSTIs, reducing their absorption. Integrase strand transfer inhibitors can be taken 2 h before or 6 h after polyvalent cations. However, calcium and iron can be co-administered with InSTIs if taken with a meal, but not in the fasted state. Integrase strand transfer inhibitors are victim drugs when co-administered with strong inducers. InSTIs are not perpetrator drugs, except DTG that inhibits an efflux transporter important in the elimination of metformin (metformin dose should not exceed 500 mg 12 hourly).

There are many important pharmacokinetic drug interactions between ARVs and other drugs, as well as between different ARVs. Some of these drug–drug interactions are discussed in other sections of this article (e.g. interactions with RIF in section 18). The full list of all potential drug interactions is very long and beyond the scope of this article.

Knowledge of drug interactions is constantly evolving. Clinicians are advised to seek reliable information on drug–drug interactions when using non-standard ART regimens and when drugs are co-administered, using one or more of the resources listed in Box 4.

◦ **Common pitfalls:**
  ◦ **Not checking for interactions between concomitant drugs and current or newly initiated ARVs.** Concomitant drugs may need dose adjustment or discontinuation when ART is switched, for example, switching from a moderate inducer (EFV) to a strong inhibitor (PI/r), or from either of these to an InSTI.
  ◦ **Not considering marked increases in statin considerations when used concomitantly with PIs.** There are major interactions between PIs and many statins, which result in marked increases in statin concentrations. Low-dose atorvastatin (not exceeding 10 mg, which will give an equivalent exposure to about 60 mg) can be used with PIs, but simvastatin cannot be used.

BOX 4 Contacts and resources for seeking reliable information on drug–drug interactions.

- Package inserts of ARVs and concomitant drugs
- University of Liverpool HIV Drug Interactions Checker: https://www.hiv-druginteractions.org/checker
- Aid for AIDS Clinical Guidelines contain tables giving advice on drug–drug interactions: http://www.aidforaids.co.za/dp_clin.php
- University of Cape Town (UCT) Medicines Information Centre (MIC) has a regularly updated table of interactions between ARVs and drugs used in the public sector (essential medicines list): http://www.mic.uct.ac.za/sites/default/files/image_tool/images/51/EML-ART%20Interaction%20Booklet%20Feb%202019_0.pdf
- UCT MIC HIV & TB Hotline: 0800 212 506 or 021 406 6782

ARV, antiretroviral; HIV, human immunodeficiency virus; AIDS, acquired immune deficiency syndrome.

## 18. Tuberculosis

### Key points

➢ Rifampicin is a potent inducer of certain drug-metabolising enzymes and drug transporters and reduces exposure to drugs in the InSTI, NNRTI and PI classes, necessitating dose adjustments of some of these drugs.
➢ LPV/r is the only PI that can be used with RIF, but the LPV/r dose needs to be doubled.
➢ Rifabutin (RFB) can be used with all PIs, but an RFB dose adjustment is required.
➢ Several side effects are shared between ARVs and TB drugs, including GI intolerance, hepatotoxicity, drug rashes, myelosuppression and neuropsychiatric side effects.

### Considerations for antiretroviral therapy in the context of tuberculosis

Tuberculosis is the most frequent co-infection affecting HIV-positive people in southern Africa. Patients may be diagnosed with TB at entry or re-entry into HIV care, or diagnosed with active TB whilst on ART. Studies in South Africa have suggested that TB incidence remains higher in patients who are virally suppressed on long-term ART compared with HIV-negative people living in the same community, possibly because of persisting defects in anti-mycobacterial immunity. The co-treatment of HIV and TB is complex because of (1) drug–drug interactions (discussed below), (2) TB-IRIS (section 26) and (3) shared side effects (discussed below). These issues, which have recently been reviewed,^80^ affect decisions regarding the timing of ART in ART-naive patients with TB (section 6).

Certain ART regimens need to be modified for compatibility with RIF. Rifampicin is a critical component of the drug-sensitive TB regimen that substantially reduces the risk of relapse after completing TB treatment.

There are no significant interactions between NRTIs and RIF; however, InSTIs, NNRTIs, PIs and MVC all exhibit drug interactions with RIF. Dolutegravir can be used in patients receiving RIF, but a dose adjustment is required (Table 19).^81^ Efavirenz is the preferred NNRTI for use with RIF. Nevirapine was previously recommended as an alternative in patients with contraindications to EFV (e.g. psychosis), but it carries a higher risk of virological failure when used with RIF, and given the availability of the InSTI class, NVP is no longer recommended. Rilpivirine and ETR cannot be used with RIF. The plasma concentrations of all PI/r are reduced to subtherapeutic ranges with RIF. Dose adjustment of LPV/r can overcome this induction (Table 19), but there is a risk of hepatotoxicity; patients require counselling and ALT should be monitored frequently.^82,83^

**TABLE 19:** Antiretroviral drug interactions with rifampicin and recommendations for co-administration.

Class	ART drug	Interaction	Dose of ART drug with RIF
NRTI	All in class	No significant pharmacokinetic interactions	No dose adjustment required
NNRTI	EFV	Mild reduction in EFV concentrations INH increases EFV concentrations in genetic slow metabolisers (~20% of South Africans), who already have high EFV concentrations – this can result in toxicity	No dose adjustment required (600 mg nocte)
	ETR and RPV	Marked reduction in concentrations	Do not prescribe concomitantly with RIF
PI/r	LPV/r	LPV plasma concentrations significantly decreased	Double the dose of LPV/r to 800 mg/200 mg 12 hourly There is an increased risk of hepatotoxicity with this strategy. The dose adjustment can be made gradually over 1–2 weeks. Dose adjustment should be continued for 2 weeks after RIF is stopped
	All other PI/r	Significant reduction in PI concentrations	Do not prescribe concomitantly
InSTI	RAL	Reduction in concentrations	Increase dose to 800 mg 12 hourly
	DTG	Significant reduction in concentrations	Increase dose frequency to 50 mg 12 hourly
CCR5 blocker	MVC	Reduction in concentrations	Increase MVC dose to 600 mg twice daily when co-administered with RIF in the absence of a potent CYP3A4 inhibitor

ART, antiretroviral therapy; DTG, dolutegravir; EFV, efavirenz; ETR, etravirine; InSTI, integrase inhibitor (integrase strand transfer inhibitor); LPV, lopinavir; LPV/r, ritonavir-boosted lopinavir; MVC, maraviroc; NNRTI, non-nucleoside reverse transcriptase inhibitor; NRTI, nucleoside reverse transcriptase inhibitor; PI, protease inhibitor; PI/r, ritonavir-boosted protease inhibitors; RAL, raltegravir; RIF, rifampicin; RPV, rilpivirine; INH, isoniazid.

An alternative approach is to replace RIF with RFB in patients taking a PI/r. However, RFB is not co-formulated with other TB drugs, and the evidence base for RFB in the treatment of TB is much less substantial than that for RIF.^84^ There is also uncertainty regarding the optimal dose of RFB with PI/r; these guidelines recommend 150 mg daily (Table 20) for efficacy reasons, but careful monitoring for toxicity is required (ALT, neutrophil count and visual symptoms at least monthly).^85^ RFB may be considered in patients who are not able to tolerate co-treatment with double-dose LPV/r and RIF-based TB treatment (i.e. patients unable to tolerate the increased LPV/r dose because of hepatotoxicity or GI side effects) or in ART-experienced patients on an ART regimen that is not compatible with RIF (e.g. third-line ART with DRV/r). If RFB is unavailable and adjusted doses of LPV/r are poorly tolerated in patients receiving second-line ART, then DTG (50 mg 12 hourly) may be substituted for the PI. However, it should be noted that good evidence is lacking regarding the robustness of DTG in second-line therapy with both NRTIs compromised, as exists for PI/r (section 13). Nevertheless, the short-term use of DTG with two compromised NRTIs over 6 months is preferable to treating TB without RIF, which has a high risk of failure or relapse.

**TABLE 20:** Dosage of antiretroviral drugs and rifabutin when prescribed concomitantly.

ART drug	ART dosage	RFB dosage
EFV	No change	Increase to 450 mg/day
InSTI class	No change	300 mg/day
ATV or PI/r	No change	Decrease to 150 mg/day (monitor ALT, neutrophils and visual symptoms at least monthly)
RPV	Do not co-administer or increase RPV to 50 mg daily	300 mg/day (or 150 mg/day with PI/r)
ETR	Preferably avoid, but if used, then administer standard doses of ETR	300 mg/day (or 150 mg/day with PI/r)

ALT, alanine transaminase; ART, antiretroviral therapy; ATV, atazanavir; EFV, efavirenz; ETR, etravirine; InSTI, integrase strand transfer inhibitor; PI/r, ritonavir-boosted protease inhibitor; RFB, rifabutin; RPV, rilpivirine; RTV, ritonavir.

Antiretroviral therapy and TB medication share many side effects (Table 21).

◦ **Common pitfalls:**
  ◦ **Rifampicin is co-administered with LPV/r, but the dose of LPV/r is not adjusted.** This results in sub-therapeutic LPV concentrations and development of PI resistance. Rifampicin should not be co-administered with ATV/r or DRV/r at all.
  ◦ **Combining linezolid and AZT.** These drugs should not be combined because both can cause bone marrow suppression (especially anaemia and neutropenia).

**TABLE 21:** Shared side effects ofantiretroviral therapy and tuberculosis treatment.

Side effects	ART	TB treatment
Nausea	AZT, ddI and PIs	Pyrazinamide, ethionamide and PAS
Hepatitis	EFV and PIs (NRTIs can cause steatohepatitis)	RIF, RFB, INH, pyrazinamide, bedaquiline and many second-line drugs, including quinolones
Renal impairment	TDF	Aminoglycosides and RIF (rare)
Rash	EFV, RAL and DTG	RIF, RFB, INH, pyrazinamide, ethambutol, streptomycin and many second-line drugs, including quinolones
Neuropsychiatric complications	EFV and DTG	Terizidone/cycloserine, quinolones, INH
Prolonged QTc	RPV	Bedaquiline, quinolones, clofazimine and delamanid
Myelosuppression	AZT	RFB and linezolid

ART, antiretroviral therapy; AZT, zidovudine; d4T, stavudine; ddI, didanosine; DTG, dolutegravir; EFV, efavirenz; INH, isoniazid; NRTIs, nucleoside reverse transcriptase inhibitors; PIs, protease inhibitors; RAL, raltegravir; RFB, rifabutin; RIF, rifampicin; TB, tuberculosis; TDF, tenofovir; QTc, corrected QT interval; RPV, rilpivirine; PAS, p-aminosalicylic acid.

## 19. Pregnancy and breastfeeding

**Note:** It is beyond the scope of these guidelines to provide comprehensive guidance for the management of pregnant women. Key recommendations relating to the mother are included, but providers are encouraged to refer to national guidelines. All women should be linked to routine antenatal care when pregnancy is confirmed.

- The prevention of mother-to-child transmission of HIV (PMTCT) programme includes periconception, pregnancy, delivery and breastfeeding and encompasses the prevention of unplanned pregnancies.
- In low-resource settings, breastfeeding commonly continues for up to 24 months. Breastfeeding transmission is now the most common mode of mother-to-child transmission of HIV in many parts of sub-Saharan Africa, rendering post-natal retention-in-care vital to successful PMTCT intervention.
- Virological suppression on ART is essential for maternal health, and to prevent HIV transmission to the infant. An elevated VL > 50 copies/mL in a pregnant or breastfeeding woman requires urgent action.
- In well-functioning PMTCT programmes, a significant proportion of infections in infants result from undetected seroconversion during pregnancy and breastfeeding. Repeated HIV testing throughout these periods is essential for women initially testing HIV-negative.
- Interventions that support HIV risk reduction in women include male partner HIV testing and linkage to ART for ‘treatment as prevention’, encouraging consistent condom use throughout pregnancy and breastfeeding, and providing PrEP to women who are at substantial risk of HIV infection.
- Maternal health is central to healthy infants, and is an essential focus of PMTCT services: advanced HIV results in life-threatening OIs, leading to miscarriage, stillbirth, premature delivery and maternal death.

### Mother-to-child transmission of human immunodeficiency virus

Overall, the risk of mother-to-child transmission of HIV is ~ 40% in the absence of any intervention (see Box 5 for more information). Timing of such transmission is as follows: *in utero*: 5%; during delivery: 15% – 20%; up to 24 months of breastfeeding: 20%.

BOX 5 South African national guidelines for the prevention of mother-to-child transmission of human immunodeficiency virus.

South African National Department of Health. Guideline for the Prevention of Mother to Child Transmission of Communicable Infections. Pretoria, South Africa: National Department of Health, 2019.

https://sahivsoc.org/Files/PMTCT%20Guideline%20November%20signed%20PRINT%20v7.pdf

South African National Department of Health. 2019 ART Clinical Guidelines for the Management of HIV in Adults, Pregnancy, Adolescents, Children, Infants and Neonates. Pretoria, South Africa: National Department of Health, 2019.

https://sahivsoc.org/Files/2019%20Abridged%20ART%20Guidelines%2010%20October%202019.pdf

The time of the highest risk coincides with delivery, which spans a matter of hours; the risk during 24 months of breastfeeding is slightly higher, but over a significantly greater timespan. Breastfeeding should not be stopped because of a new diagnosis of HIV, or an elevated VL in women already on ART. Instead, initiation of ART and management of raised VL (together with infant prophylaxis) are interventions to ‘make breastfeeding safer’.

### Antiretroviral therapy for women of childbearing potential and during pregnancy and breastfeeding

All HIV-positive pregnant and breastfeeding women should be initiated on lifelong ART, ideally the same day that pregnancy is confirmed. Standard first-, second- and third-line regimens should be used in pregnancy (see the sections ‘Initial antiretroviral therapy regimens for the previously untreated patient’, ‘Management of patients currently receiving first-line therapy’, ‘Management of patients starting or currently receiving second-line therapy’ and ‘Third-line antiretroviral therapy’). Regarding DTG use in pregnancy, it is important to note that the absolute risk of NTD is low (< 0.5%), and this risk may be outweighed by the additional benefits of DTG over alternative therapies. We currently recommend that WOCP who wish to become pregnant or who have no reliable access to effective contraception should be counselled adequately about the potential risks and benefits of DTG- versus EFV-based ART, and should be offered the choice of first-line regimens.

Other points regarding ART in pregnancy include the following:

- Efavirenz 600 mg is a safe and effective regimen for use by WOCP including during the time from conception to the end of the first trimester. There are insufficient data to recommend routine use of EFV 400 mg in pregnant women.
- The current guidelines no longer recommend initiating NVP in any patients. Maternal deaths in pregnant women have been associated with NVP because of liver and skin hypersensitivity reactions.
- NRTIs: Note that commonly used CrCl calculations are not validated for pregnant women; therefore, avoid TDF if serum Cr ≥ 85 µmol/L.
- Dose adjustment of ART during pregnancy is only indicated for women taking both TDF and ATV/r during the second/third trimester; the dose should be increased from ATV/r 300 mg/100 mg to 400 mg/100 mg.
- Women taking LPV/r 800 mg/200 mg daily should be advised to adjust this to 400 mg/100 mg 12 hourly (twice daily) during pregnancy because of altered pharmacokinetics. These women should also be informed about the association between LPV/r and premature labour and delivery.
- Particular importance should be placed on drug–drug interactions between DTG and divalent cation-containing medication in pregnancy, as pregnant women frequently receive iron supplements and/or magnesium-/aluminium-containing antacids.

Patients returning to care in pregnancy after defaulting a first-line regimen or those exposed to previous PMTCT regimens should generally be put directly on a DTG-based regimen, rather than retrying an NNRTI-regimen (section 16). As per current PMTCT guidelines, women not already on ART at the time of labour or delivery should commence TLD immediately and also receive an additional stat dose of NVP 200 mg. Women who are newly diagnosed with HIV during the breastfeeding period may continue breastfeeding as per maternal preference, provided that maternal ART and infant prophylaxis are initiated and adherence support is given.

Other key recommendations:

- All pregnant women should be screened at every visit for sexually transmitted infections and treated as needed.
- All pregnant and breastfeeding women should be screened for TB at every visit. If the TB screening is negative, then consider TB-preventive therapy during pregnancy only in women with a CD4^+^ count < 350 cells/µL (section 27).

◦ **Common pitfalls:**
  ◦ **Not performing VL monitoring at appropriate time.** See Table 22 for the appropriate monitoring intervals.
  ◦ **An elevated VL is not acted upon urgently.** Viral load results should be fast-tracked, and women failing their current regimen must be identified early and, if necessary, a regimen switch should be made without delay.

**TABLE 22:** Timing of viral load monitoring during pregnancy, delivery and breastfeeding.

Variable	ART initiation in pregnancy	Already on ART at diagnosis of pregnancy	Previously taken ART, not currently on treatment (ART interruption, ART for PMTCT)	Newly diagnosed HIV infection during delivery or breastfeeding
Antenatal	VL at baseline and after 3 months of ART: if > 28 weeks’ gestation, then repeat VL at delivery	VL at first ANC visit	VL at initiation of DTG-based regimen; repeat VL 3 months later – change in VL determines management	-
Delivery	All women need VL measurement at delivery; review result at day 3–6 postnatal visit	All women need VL measurement at delivery; review result at day 3–6 postnatal visit	All women need VL measurement at delivery; review result at day 3–6 postnatal visit	-
Postnatal, up to the end of breastfeeding	VL measure at 6 months postpartum; repeat VL 6 monthly during breastfeeding	VL measure at 6 months postpartum; repeat VL 6 monthly during breastfeeding	VL measure at 6 months postpartum; repeat VL 6 monthly during breastfeeding	VL after 3 months of ART, then 6 monthly during breastfeeding

ANC, antenatal care; ART, antiretroviral therapy; DTG, dolutegravir; PMTCT, prevention of mother-to-child transmission; VL, viral load; HIV, human immunodeficiency virus.

## 20. Liver disease

### Antiretroviral dose adjustment in liver impairment

#### Key points

➢ There is no single blood test for accurate quantification of liver impairment.
➢ Child–Pugh class C may require dose adjustment for some ART drugs.
➢ The combination of TDF + 3TC (or FTC) + DTG (or RAL) is regarded as least hepatotoxic.

#### Antiretroviral dose adjustments

Table 23 shows the outline of dose adjustments for the relevant ART drugs in patients with Child–Pugh class C liver impairment.

**TABLE 23:** Prescribing antiretroviral therapy in liver impairment.

Class	Drug	Prescribing notes
NRTI	TDF	No dose adjustment necessary
	3TC	No dose adjustment necessary
	FTC	No dose adjustment necessary
	AZT	Decrease dose by 50% or double dosage interval in significant liver disease
	ABC	Reduce adult dose to 200 mg twice daily in significant liver disease Contraindicated in severe liver disease
InSTI	DTG	No data on recommendation for those with severe liver disease (Child–Pugh class C)
	RAL	No dose adjustment necessary
PI	DRV	Use with caution or avoid in significant liver disease
	ATV	Avoid in severe liver disease
	LPV/r	LPV is highly metabolised in the liver and concentrations may be increased in patients with hepatic impairment Therapeutic drug monitoring should be done if available
NNRTI	EFV	Not recommended in severe liver disease
	ETR	Use with caution in severe liver disease
	RPV	Use with caution in severe liver disease (Child–Pugh class C) – dose recommendation not established
CCR5 blocker	MVC	Concentrations likely to be increased with liver impairment

3TC, lamivudine; ABC, abacavir; ATV, atazanavir; ARVs, antiretrovirals; AZT, zidovudine; CCR5, C-C chemokine receptor type 5; DTG, dolutegravir; DRV, darunavir; EFV, efavirenz; ETR, etravirine; FTC, emtricitabine; InSTI, integrase strand transfer inhibitor; LPV, lopinavir; LPV/r, lopinavir/ritonavir; MVC, maraviroc; NNRTI, non-nucleoside reverse transcriptase inhibitors; NRTI, nucleoside reverse transcriptase inhibitors; PI, protease inhibitor; RAL, raltegravir; RPV, rilpivirine; TDF, tenofovir disoproxil fumarate.

### Hepatitis B and human immunodeficiency virus co-infection

#### Key points

➢ All HIV-infected individuals should be screened for active HBV – hepatitis B surface antigen (HBsAg) screening is an appropriate test.
➢ The HBV VL correlates with disease progression and is used to monitor anti-HBV therapy.
➢ All children and adults eligible for HBV vaccination should be vaccinated.
➢ Antiretroviral therapy drugs with anti-HBV activity are TDF + 3TC (or FTC).
➢ For all HIV-infected HBsAg-positive patients, the ART regimen should include TDF + 3TC (or FTC).
➢ Using 3TC without TDF to treat HBV/HIV co-infection leads to HBV resistance in 80% – 90% of patients after 5 years of treatment.
➢ Interruption of TDF and/or 3TC (or FTC) has been associated with flares of life-threatening hepatitis in patients with hepatitis B in case reports.
➢ Adjust dosing frequency of TDF in patients with HBV infection and renal dysfunction; if renal function is severe or deteriorates with TDF, then 3TC monotherapy or other drugs with anti-HBV activity should be considered.

Hepatitis B virus is a common co-infection with HIV in southern Africa, with significant implications for progression to cirrhosis, as well as for treatment options. Access to vaccination, laboratory resources and treatment options is limited to some extent in southern African countries, and the recommendations below should be considered in the light of the local context.

Hepatitis B virus and HIV co-infection is associated with:

- an increased risk of chronic liver disease
- a higher HBV VL
- diminished responses to HBV vaccine
- an increased risk of drug-induced hepatotoxicity
- a flare of hepatitis within 3 months of commencing ART (because of HBV-related IRIS, which is difficult to differentiate from drug hepatotoxicity).

Drugs directed against HBV that have no or minimal anti-HIV activity (e.g. entecavir and telbivudine) are largely unavailable or extremely expensive in southern African region. Instead, it is usually necessary to use ART drugs that also have anti-HBV activity: TDF + 3TC (or FTC). As with HIV, these drugs suppress HBV but do not eradicate it. Effective treatment prevents or slows the progression to cirrhosis. For all HIV-infected HBsAg-positive patients, the ART regimen should include TDF + 3TC (or FTC). Using 3TC without including TDF leads to the development of HBV resistance in 80% – 90% of patients after 5 years of treatment. If a patient meets the criteria for switching to a second-line ART regimen (to treat HIV), then this combination – TDF + 3TC (or FTC) – should be continued to suppress HBV infection, as interruption of TDF and/or 3TC (or FTC) has been associated with flares of life-threatening hepatitis in case reports. The second-line ART regimen should be shaped around these two drugs.

In patients with HBV and renal dysfunction, the use of TDF may be considered with dosing frequency adjustment based on CrCl (Table 24) and more frequent creatinine monitoring. If renal dysfunction is severe or renal function deteriorates with TDF, then 3TC monotherapy or other drugs with anti-HBV activity should be considered.

◦ **Common pitfalls:**
  ◦ **Not continuing with TDF + 3TC (or FTC) combination when switching to second-line ART.** The second-line ART regimen should be shaped around these two drugs.
  ◦ **Using 3TC without including TDF in the treatment of HIV/HBV co-infected patients.**

**TABLE 24:** Suggested tenofovir disoproxil fumarate dose adjustment in patients with hepatitis B and renal dysfunction.

eGFR (mL/min/1.73 m^2^)	Suggested dose of TDF
≥ 50	Usual dose
30–49	300 mg every 48 h
10–29	300 mg every 72–96 h
< 10	Avoid (consider 3TC monotherapy, or other drugs with anti-HBV activity)
Haemodialysis	300 mg every week (dose after dialysis on dialysis days)

3TC, lamivudine; eGFR, estimated glomerular filtration rate; HBV, hepatitis B virus; TDF, tenofovir disoproxil fumarate.

## 21. Renal disease

### Antiretroviral drug dose adjustment in renal disease

#### Key points

➢ Renal function is estimated by the modified Cockgraft–Gault formula or modification of diet in renal disease (MDRD) formula.
➢ For haemodialysis, the ART prescribed should be taken after dialysis.

In HIV-positive patients on chronic haemodialysis, there are a number of important ART considerations. The NRTI class is eliminated through the kidneys; thus, most NRTIs require dose adjustment as shown in Table 25.^86,87,88^ For suggested TDF dosing in patients with chronic hepatitis B, (section 20).

**TABLE 25:** Antiretroviral drug dose adjustments in renal failure.^86,87,88^

Drug	CrCl†,§		Haemodialysis (dosage after dialysis)	Peritoneal dialysis
	10–50 mL/min	< 10 mL/min		
TDF	Avoid	Avoid	300 mg once weekly	Unknown
ABC	Unchanged	Unchanged	Unchanged	Unchanged
3TC	150 mg daily	50 mg daily¶	50 mg first dose, thereafter 25 mg daily¶	50 mg first dose, thereafter 25 mg daily¶
AZT	Unchanged	300 mg daily	300 mg daily	300 mg daily
NNRTIs	Unchanged	Unchanged	Unchanged	Unchanged
PIs	Unchanged	Unchanged	Unchanged	Unchanged
InSTIs	Unchanged	Unchanged	Unchanged	Unchanged

*Source*: Bartlett JG, Gallant JE, Pham PA (eds). Medical management of HIV infection. 15th ed. Baltimore, MD: John Hopkins University Press, 2009–2010; 556 pp; Gilbert DN, Moellering RC, Eliopoulos GM, et al. (eds). The Sanford Guide to antimicrobial therapy. 42nd ed. Sperryville, Virginia: Antimicrobial Therapy, Inc., 2012; 232 pp; HIV Medicine Association of the Infectious Diseases Society of America. Clinical practice guideline for the management of chronic kidney disease in patients infected with HIV: 2014 update. Clin Infect Dis. 2014;59(9):e96–e138.

3TC, lamivudine; ABC, abacavir; ART, antiretroviral therapy; AZT, zidovudine; CrCl, creatinine clearance rate; d4T, stavudine; ddI, didanosine; eGFR, estimated glomerular filtration rate; InSTIs, integrase strand transfer inhibitors; MDRD, modification of diet in renal disease; NNRTIs, non-nucleoside reverse transcriptase inhibitors; PIs, protease inhibitors; NRTIs, nucleoside reverse transcriptase inhibitors; sCr, serum creatinine; TDF, tenofovir disoproxil fumarate.

† , Many laboratories report the eGFR calculated using a variation of the MDRD formula. This result can be used (in place of the calculated CrCl) to make decisions regarding the use of TDF and for modification of the dose of other NRTIs based on this table.

¶ , Some experts recommend that the lowest available tablet dose of 150 mg 3TC daily should be used in patients with advanced renal disease (CrCl < 10 mL/min) and patients on dialysis so as to avoid having to use the liquid formulation of 3TC, and because of the favourable safety profile and lack of data to suggest 3TC dose-related toxicity. This is particularly relevant if the 3TC liquid formulation is unavailable or not tolerated.

§ , The modified Cockgraft–Gault equation: CrCl = (140 – age × ideal weight) ÷ sCr. For women, multiply the total by 0.85.

### Antiretroviral drug choice and dosing in patients on chronic haemodialysis

#### Key points

➢ Patients with HIV may develop end-stage renal failure owing to HIV-associated nephropathy or an HIV-unrelated cause, necessitating chronic haemodialysis.
➢ Tenofovir disoproxil fumarate can be used in patients on chronic haemodialysis, but with once-weekly dosing which can be difficult for patients to remember.
➢ Zidovudine is generally avoided because of anaemia associated with renal failure.
➢ Integrase strand transfer inhibitors; and NNRTI drugs do not require dose adjustment.
➢ Atazanavir concentrations are reduced in patients on haemodialysis to a greater extent than LPV concentrations.
➢ Lopinavir/ritonavir requires a twice-daily dosing in patients on haemodialysis.
➢ Antiretroviral therapy drugs taken once daily, or the evening doses of drugs taken twice daily, should be given after haemodialysis session on dialysis days to prevent the drug from being dialysed out.
➢ Patients on chronic haemodialysis should be reviewed by a clinician experienced in ART management at least 6 monthly to monitor treatment efficacy and side effects and to adjust the regimen when needed.

#### Recommendations for antiretroviral therapy for patients on chronic haemodialysis

We recommend the following first-line option for patients on chronic haemodialysis: ABC (600 mg daily) + 3TC (50 mg first dose and thereafter 25 mg daily) + DTG (50 mg daily). On the days when haemodialysis is performed, the drugs should be given after the haemodialysis session.

- **Common pitfall: Not giving daily doses or the evening doses of a twice-daily regimen after the haemodialysis session on dialysis days to prevent the drug from being dialysed out.**

### Antiretroviral therapy in patients with acute kidney injury

#### Key points

➢ In patients with AKI, NRTI dose adjustments should be implemented based on estimated CrCl calculation.
➢ Tenofovir disoproxil fumarate should be interrupted even if it is not thought to be the cause of the AKI.
➢ Re-challenge with TDF may be considered in patients 1-month post-resolution of AKI if TDF was not the cause and renal function returns to normal.
➢ In patients with AKI who have not yet received ART, initiation is preferably deferred until AKI has resolved. But avoid significant delays.
➢ Once renal function improves (i.e. creatinine is on a downward trend), standard NRTI doses should be re-introduced to avoid under-dosing.

#### Antiretroviral therapy in acute kidney injury

In patients with AKI, NRTI dose adjustments should be implemented based on estimated CrCl calculation (see Table 25). Tenofovir disoproxil fumarate should be interrupted even if it is not thought to be the cause of AKI. Care should be taken to identify other drugs that may affect renal function, such as aminoglycoside antibiotics, non-steroidal anti-inflammatory drugs, cotrimoxazole and iodinated radiocontrast; these drugs should be avoided where possible, including temporary discontinuation.

Once there is clear evidence that renal function is improving (i.e. creatinine is on a downward trend), standard NRTI doses should be reintroduced to avoid under-dosing. In patients with AKI who have not yet received ART, initiation is preferably deferred until AKI has resolved. However, ART should not be delayed more than 2 weeks for this reason. If renal function does not show any improvement after treating an acute event (e.g. sepsis) and this persists beyond 3 months, then the patient should be assessed for chronic kidney disease and referred to a physician who can evaluate and investigate further.

◦ **Common pitfall: Not performing NRTI dose adjustment in patients with AKI.**

## 22. Psychiatric disease

### Key points

➢ Dolutegravir may cause insomnia, headache and neuropsychiatric side effects.
➢ Zidovudine and RAL frequently cause headaches when started, but this usually resolves.
➢ The majority of patients who experience neuropsychiatric features of EFV do so within the first 2–6 weeks, and thereafter the drug is better tolerated. Late neurological syndromes are described however (see section 4).
➢ Most neuropsychiatric effects relating to ART occur in the first few weeks of therapy.
➢ Depression and other mental illnesses are often undiagnosed or undertreated in HIV-infected individuals and may undermine adherence.
➢ Consider avoiding EFV- and RPV-based regimens in patients with psychiatric illness – these drugs can exacerbate psychiatric symptoms and may be associated with suicidality.

Mental health, especially mood and behaviour disorder, is associated with non-adherence to ART, leading to disability and poorer HIV treatment outcomes. There is a higher prevalence of depression in HIV-positive individuals, with a reported range of 20% – 40% versus 10% in the general population.^89^

Zidovudine and RAL frequently cause headaches when started, but this usually resolves after sometime. Efavirenz frequently causes neuropsychiatric effects in the first few weeks of therapy, typically presenting with insomnia, vivid dreams and dizziness. Both dysphoria and euphoria may occur. Fortunately, these features subside in the majority of patients within the first 4–6 weeks. Psychosis may occasionally occur. Dolutegravir may cause insomnia, headache and neuropsychiatric side effects. Raltegravir has been associated with similar central nervous system side effects.

◦ **Common pitfalls:**
  ◦ **Not warning patients starting ART about potential neuropsychiatric symptoms.** Patients must be informed about potential side effects.
  ◦ **Unnecessary delays in initiating ART in patients with psychiatric illness.**

## 23. Malaria

### Key points

➢ There are several drug interactions between antimalarial agents and ART drugs.
➢ Efavirenz has a significant drug interaction with artemether–lumefantrine (Coartem) such that artemether (and its active metabolite) and lumefantrine concentrations are lowered, which can lead to failure of antimalarial therapy. Consider extending the course of artemether–lumefantrine to 6 days if administered concurrently with EFV.
➢ No artemether–lumefantrine dose adjustment is recommended for patients taking PIs or InSTIs.
➢ Protease inhibitors and NNRTIs exhibit several interactions with atovaquone–proguanil (Malanil) such that atovaquone concentrations are reduced – atovaquone–proguanil (Malanil) is best avoided in patients receiving these drugs.
➢ No significant drug interactions are predicted between InSTIs (DTG) and antimalarial drugs.
➢ Quinine is best avoided in patients on PIs or NNRTIs.

There are several drug interactions between antimalarials and ART drugs (see Table 26). Whilst artemether–lumefantrine (Coartem) can be administered safely with NVP, EFV significantly lowers the concentrations of artemether (and its active metabolite) and lumefantrine, which is likely to increase the risk of failure of antimalarial therapy. There is no clear guideline on how to overcome this interaction, but some experts recommend repeating the 3-day course of artemether–lumefantrine (i.e. treat for 6 days). Boosted PIs dramatically increase the plasma concentrations of lumefantrine, but a dose reduction is not recommended, as the toxicity threshold of lumefantrine seems to be high. Close monitoring for toxicity is recommended when co-administering artemether–lumefantrine with ART.

**TABLE 26:** Important drug–drug interactions between antimalarial agents and antiretroviral therapy drugs.

Drug	Antimalarial agent	Direction of interaction	Recommendation
InSTI	Coartem (artemether–lumefantrine)	No interaction	Safe to use
	Atovaquone–proguanil	No interaction	Safe to use
EFV	Coartem	↓ Artemether and lumefantrine concentrations	Use but might need to repeat the 3-day course of Coartem
	Atovaquone–proguanil	↓ Atovaquone and proguanil concentrations	Avoid co-administration
NVP	Coartem	No interaction	Use
	Atovaquone–proguanil	↓ Atovaquone concentrations	Avoid co-administration
PI/r	Coartem	↑ Lumefantrine concentrations	No dose adjustment necessary
	Atovaquone–proguanil	↓Atovaquone and proguanil concentrations	Avoid co-administration

ART, antiretroviral therapy; EFV, efavirenz; InSTI, integrase strand transfer inhibitor; NVP, nevirapine; PI/r, ritonavir-boosted protease inhibitor.

Quinine concentrations are significantly decreased by LPV/r, probably owing to induction of metabolism by RTV. It is likely that quinine concentrations will also be reduced by EFV and NVP; therefore, quinine should be avoided in patients receiving PIs or NNRTIs. Patients with severe malaria should receive artesunate, if available, and those with milder malaria should be treated with artemether–lumefantrine.

Amongst drugs used for chemoprophylaxis, there are no clinically significant pharmacokinetic interactions between ARVs and mefloquine or doxycycline. However, mefloquine and EFV both cause frequent neuropsychiatric side effects; therefore, doxycycline is the preferred chemoprophylactic agent for patients receiving EFV.

There are several interactions with atovaquone–proguanil (Malanil). Atovaquone concentrations are reduced by PIs and EFV, and also likely by NVP. Proguanil concentrations are also reduced by PIs and EFV. The use of atovaquone–proguanil is therefore best avoided in patients receiving PIs or NNRTIs.

No significant drug interactions are predicted between InSTIs and antimalarial drugs.

◦ **Common pitfalls:**
  ◦ Not advising patients receiving ART on chemoprophylaxis for malaria when travelling to malaria-endemic areas.
  ◦ **Not providing ART recipients with intravenous artesunate or Coartem for malaria treatment despite the potential drug interactions.**

## 24. Antiretroviral drug-induced liver injury

### Key points

➢ All ART classes have been associated with hepatotoxicity and cause injury through an idiosyncratic reaction as the mechanism of injury.
➢ Alanine transaminase elevations greater than five times the upper limit of normal (ULN) are significant in the absence of symptoms.
➢ In the presence of symptoms of hepatitis, ALT elevations greater than 2.5 times the ULN are significant.
➢ Nevirapine is most frequently associated with DILI, with most cases occurring in the first few months after initiation. We no longer recommend NVP initiation.
➢ Patients on EFV may present with a delayed DILI many months after commencing therapy.
➢ Re-challenge is best avoided and may be considered in select cases in consultation with a specialist.
➢ If severe hepatitis occurs, or any hepatitis together with a rash, fever or systemic reaction occurs, then re-challenge with
➢ NNRTIs, ABC or CTX should not be attempted.

An ALT test should be performed in all patients at ART initiation. Repeat ALT testing is indicated in those who develop symptoms or signs suggestive of hepatitis. All ARV classes have been associated with hepatotoxicity – most commonly NNRTIs. Mild ALT elevations occur commonly and in general are transient. Alanine transaminase elevations greater than five times the ULN are significant in the absence of symptoms. In the presence of symptoms of hepatitis, ALT elevations greater than 2.5 times the ULN are also significant. In such patients, potentially hepatotoxic ARVs should be switched to alternative agents. Management guidelines are provided in Table 27.

**TABLE 27:** Guidelines for managing hepatotoxicity.

Elevation	ULN†		
	< 2.5 × ULN	2.5 − 5 × ULN	> 5 × ULN
ALT	Repeat at 1–2 weeks	Repeat at 1 week	Discontinue relevant drug(s)
Bilirubin	Repeat at 1 week	Discontinue relevant drug(s)	Discontinue relevant drug(s)

ALT, alanine transaminase; ULN, upper limit of normal.

† , Any elevations with symptoms of hepatitis (nausea, vomiting and right upper quadrant pain) should be regarded as an indication to discontinue the relevant drugs.

Re-challenge may be considered, and in selected cases a specialist should be consulted. If severe hepatitis occurs, or any hepatitis with rash, fever or other systemic manifestation occurs, the opinion of a specialist should be sought. In this situation, re-challenge with NNRTIs, ABC or CTX should not be attempted.

Prolonged use of NRTIs, especially d4T and ddI (both of which are no longer used), may cause fatty liver. Typically, ALT concentration is more significantly elevated than aspartate transaminase (AST), and the concentrations of canalicular enzymes gamma-glutamyl transferase (GGT) and alkaline phosphatase (ALP) are more elevated than those of the transaminases. Non-tender hepatomegaly may be present. Ultrasound or computed tomography (CT) imaging may show decreased hepatic density. The condition is not benign, and fibrosis has been reported with long-term ddI use. Patients should be advised to avoid alcohol and should be switched to alternative drugs with lower potential for causing fatty liver.

In patients with severe hepatitis or jaundice, features of hepatic encephalopathy (i.e. features of hepatic failure) must be clinically assessed, and the international normalised ratio (INR) and serum glucose should be checked.

If the concentration of canalicular enzymes is more significantly elevated than that of ALT, or if conjugated bilirubin is elevated, then an ultrasound of the liver should be conducted to exclude biliary obstruction.

Isolated unconjugated hyperbilirubinaemia (drug-induced Gilbert’s syndrome) is associated with ATV. In this case, all other LFTs are normal and the patient has no other symptoms of hepatitis. Although this is a benign condition (it does not reflect liver injury, but isolated competitive inhibition of the enzyme in the liver which conjugates bilirubin), it is often cosmetically unacceptable to patients, necessitating a switch from ATV to an alternative drug.

Although EFV has been recognised as an infrequent cause of DILI since it first became available, a novel pattern of DILI associated with the drug has recently been recognised in South Africa.^25^ Amongst such patients, many had a particularly severe pattern of liver injury found on liver biopsy (termed ‘submassive necrosis’, and associated with severe jaundice and a raised INR). The overall mortality was 11%. Whilst severe EFV-related DILI is likely to be uncommon, clinicians should be aware of the features observed:

1. The diagnosis of DILI was generally made after a longer duration on EFV (~ 3–6 months) than what is seen with DILI related to NVP or TB medication.
2. The DILI was not associated with features of hypersensitivity (e.g. drug rash) and often the first symptom was jaundice rather than abdominal symptoms.
3. Once EFV was stopped, it typically took several months for LFTs to normalise (median resolution > 6 months).

*We do not advise routine LFT monitoring in patients on ART,* as there is no evidence that this would lead to early detection of this DILI or improve outcomes. Those managing patients on EFV should monitor for symptoms and signs of hepatitis (nausea, vomiting, right-sided abdominal pain or jaundice). If these occur, then ALT should be assessed, and the patient should be examined for jaundice. The patient should be managed appropriately for DILI if there are hepatitis symptoms with ALT > 120 U/L, or if there is jaundice. Efavirenz should be switched to an alternative drug (e.g. DTG).

Many other drugs can cause hepatotoxicity, notably anti-tuberculous agents (including prophylactic isoniazid) and azoles. Cotrimoxazole is an uncommon cause of hepatitis, often as part of a systemic hypersensitivity reaction.

Recommendations for the management of DILI in patients receiving TB treatment have been published by the Southern African HIV Clinicians Society in 2013 (see link here).^90^

◦ **Common pitfalls:**
  ◦ Failing to recognise other drugs apart from ART as a cause of hepatotoxicity.
  ◦ **Performing routine LFT monitoring in patients on ART, in an attempt to detect DILI earlier.** There is no evidence to support this approach.

## 25. Dyslipidaemia

### Key points

➢ Protease inhibitors can cause hypertriglyceridaemia and elevated LDL-C.
➢ Atazanavir/ritonavir and DRV/r are associated with less significant lipid abnormalities than LPV/r.
➢ Efavirenz can cause elevated total cholesterol and mild hypertriglyceridaemia.
➢ Dolutegravir does not significantly affect cholesterol.
➢ Lipids should be assessed routinely after 3 months on a PI regimen.

Protease inhibitors can cause hypertriglyceridaemia and elevated LDL-C. Atazanavir/ritonavir and once-daily DRV/r (800 mg DRV/100 mg once daily) are associated with less severe dyslipidaemia than other boosted PIs; AZT can cause mild hypertriglyceridaemia, and EFV can cause elevated total cholesterol and mild hypertriglyceridaemia.

We suggest that lipids should be assessed routinely after 3 months on a PI regimen. If normal at this stage, then re-assessment should be performed only in those with cardiovascular risk factors. Diet and lifestyle modification should always be advised. Diet is more effective for controlling hypertriglyceridaemia than hypercholesterolaemia. Other cardiovascular risk factors should be addressed. Clinicians should consider and investigate secondary causes of hypertriglyceridaemia and hypercholesterolaemia (e.g. diabetes, nephrotic syndrome, alcohol abuse and hypothyroidism).

If patients receiving LPV/r develop significant dyslipidaemia, they should be switched to DRV/r or ATV/r, rather than adding lipid-lowering therapy. However, lipid-lowering therapy is indicated in patients with persistent elevations despite switching to DRV/r or ATV/r. Switching the PI to DTG is another option because DTG has a more favourable lipid profile than PIs. However, DTG should only be used in a regimen in which at least one other ART drug is known to be fully active. In patients with hyperlipidaemia on EFV, the drug should be switched to DTG or RPV.

Marked hypertriglyceridaemia (> 10 mmol/L) can cause pancreatitis and requires urgent treatment with diet modification (restrict total TG intake to < 30 g/day), fibrates and switching LPV/r to DRV/r, ATV/r or DTG (fibrates can be stopped after 1 month, followed by reassessment within 4–6 weeks).

Indications for statin therapy in HIV-positive patients should be the same as in HIV-negative patients, using the Framingham heart disease risk score. As a general rule, in young patients with isolated elevated cholesterol but no other cardiovascular risk factors, a threshold of total cholesterol > 7.5 mmol/L (or LDL cholesterol > 5.0 mmol/L) should be used for initiating statin therapy, and the patient should be referred to a lipid clinic for investigation if feasible. In patients with cardiovascular risk factors (e.g. smoking, diabetes and hypertension), decisions should be made using the Framingham heart disease risk score. All patients with established atherosclerotic disease (coronary, cerebral or peripheral) or familial hypercholesterolaemia should be started on statin treatment. In addition, type 2 diabetics should also be started on a statin treatment if they have chronic kidney disease, or if they are older than 40 years of age (or have had diabetes for more than 10 years) and have one or more additional cardiovascular risk factors.^91^

Many statins have interactions with PIs that can lead to potentially toxic statin concentrations, with the exception of pravastatin and fluvastatin. Atorvastatin concentrations are significantly raised by PIs, but low doses (maximum 10 mg daily) can be used with monitoring for symptoms of myalgia. Lovastatin and simvastatin should not be co-administered with PIs, as their concentrations are dramatically increased, and severe rhabdomyolysis has been reported. We also advise against the use of rosuvastatin with PIs because of a complex drug–drug interaction; PIs increase the plasma concentrations of rosuvastatin whilst reducing their efficacy in the liver.

◦ **Common pitfalls:**
  ◦ Not routinely assessing lipids whilst the patient is receiving PI-based ART.
  ◦ Failure to recognise that many statins have interactions with PIs that lead to toxic statin concentrations.
  ◦ **Monitoring of LDL-C in patients on a high-dose statin for secondary prevention.** Such monitoring is not necessary.

## 26. Immune reconstitution inflammatory syndrome

### Key points

➢ Approximately 10% – 20% of patients who start ART with advanced immunosuppression experience IRIS in the first few months of treatment.
➢ Two forms of IRIS have been recognised, namely unmasking and paradoxical IRIS.
➢ Immune reconstitution inflammatory syndrome is most frequently described in association with TB and CM.
➢ Integrase strand transfer inhibitors are not associated with an increased risk of TB-IRIS in clinical trials.
➢ Early ART initiation (defined as 1–4 weeks after anti-tuberculous therapy) doubles the risk of TB-IRIS compared with late ART initiation (defined as 8–12 weeks after anti-tuberculous therapy), but ART should not be delayed for this reason in patients with CD4 < 50 cells/mL.
➢ There is no confirmatory diagnostic test for IRIS.
➢ In most instances, ART is continued in cases of IRIS, unless IRIS is life-threatening (e.g. neurological involvement in TB-IRIS with depressed level of consciousness).
➢ Corticosteroids have been shown to reduce morbidity and improve symptoms in paradoxical TB-IRIS.

Approximately 10% – 20% of patients who start ART with advanced immunosuppression experience clinical deterioration during the first few months because of IRIS. Most presentations of IRIS occur within the first 3 months of ART. Two forms are recognised:

- Unmasking IRIS occurs in patients who have an unrecognised OI when ART is initiated, and who then present with exaggerated inflammatory features of that infection during early ART because of it being ‘unmasked’ by recovering immunity.
- Paradoxical IRIS occurs in patients who are being treated for an OI when they start ART, but who develop an immune-mediated worsening or recurrence of features of that infection after starting ART.

Immune reconstitution inflammatory syndrome is most frequently described in association with TB and CM. Skin conditions such as molluscum contagiosum and Kaposi’s sarcoma may also worsen because of IRIS. The diagnosis of IRIS can be difficult, mainly because there is no confirmatory diagnostic test. Diagnosis relies on recognition of the characteristic clinical presentation, ensuring that OIs are correctly diagnosed, and excluding alternative causes of deterioration, such as drug resistance (e.g. multidrug-resistant TB). Case definitions for TB and cryptococcal IRIS have been published.^92,93^ It is important to ensure that the underlying OI is treated appropriately. Antiretroviral therapy should be continued, unless the IRIS is life-threatening (e.g. neurological involvement in TB-IRIS with depressed level of consciousness). Corticosteroids have been shown to reduce morbidity and improve symptoms in paradoxical TB-IRIS,^94^ and can be used in mycobacterial and fungal forms of IRIS when other causes of deterioration have been excluded, and particularly when IRIS features are severe.

For paradoxical TB-IRIS, prednisone can be commenced at a dose of 1.5 mg/kg/day and weaned over 4 weeks, but a longer course may be required if symptoms recur on weaning.^95^ Steroids should not be used in patients with Kaposi’s sarcoma.

◦ **Common pitfall: Using steroids in patients with Kaposi’s sarcoma.**

### Prophylactic prednisone

#### Key points

➢ Patients with active TB and who are improving on TB therapy with a CD4^+^ count 100 cells/mL upon starting ART can be initiated on prednisone 40 mg daily for 14 days, followed by 20 mg daily for 14 days to prevent paradoxical TB-IRIS.
➢ The use of prednisone in this context is not associated with high risk of severe infections, cancers or adverse events.

The use of prophylactic prednisone for the prevention of paradoxical TB-associated IRIS in adults with a CD4^+^ count 100 cells/mL has been shown in a randomised trial to be associated with a 30% lower relative incidence of TB-IRIS.^96^ Importantly, this did not come at the expense of any excess risk of severe infections or cancers. The recommended prednisone regimen is 40 mg daily for 14 days, followed by 20 mg daily for 14 days, and prednisone should be started concurrently with ART. Certain patient groups should be excluded from receiving prednisone; however, including patients with Kaposi’s sarcoma and patients with RIF-resistant TB, or whose TB has not improved prior to starting ART.

◦ **Common pitfall: Using prophylactic prednisone in patients who are not improving on TB therapy.**

## 27. Opportunistic infection prophylaxis

### Key points

➢ The use of appropriate prophylaxis (primary or secondary) is essential in patients initiating ART.
➢ In general, prophylaxis can be discontinued once the CD4^+^ count has increased to 200 cells/µL, but certain minimal durations of prophylaxis apply for secondary prophylaxis.
➢ Local and international guidelines should be consulted.

### Cotrimoxazole primary prophylaxis

Prophylactic CTX is indicated for HIV-positive patients with a CD4^+^ count < 200 cells/µL, or with WHO stage 3 or 4 conditions (including TB). Cotrimoxazole offers protection against *Pneumocystis jirovecii*, toxoplasmosis, isosporiasis and certain bacterial infections. The recommended dose is 160/800 mg daily. Patients who develop a hypersensitivity reaction to CTX can be given dapsone instead, although this is best avoided if the reaction to CTX is life-threatening. Cotrimoxazole can be discontinued once the patient’s CD4^+^ count *is* > 200 cells/µL.

Cotrimoxazole is a common cause of cutaneous and systemic hypersensitivity reactions, indistinguishable from hypersensitivity reactions to ART drugs. Cotrimoxazole should be interrupted when treating mild suspected NNRTI cutaneous hypersensitivity rashes, and permanently discontinued if severe hypersensitivity reactions occur. If CTX is prescribed for secondary prophylaxis or used for primary prophylaxis in those with severe immunosuppression, then an alternative should be substituted.

◦ **Common pitfall: Prescribing CTX for newly diagnosed HIV-positive patients with a high CD4**^+^ **count (> 200 cells/**m**L).**

### Cryptococcal antigen screening and pre-emptive treatment

#### Key points

➢ Cryptococcal antigenaemia screening should be performed for all adults or adolescents with a CD4^+^ count < 200 cells/mL who are initiating or re-initiating ART.
➢ Reflex laboratory screening is the preferred approach in South Africa.
➢ Lumbar puncture is recommended for all patients with a new positive CrAg screening test.

Screening for subclinical cryptococcal disease has been shown to have a benefit in reducing mortality in HIV-infected patients with a CD4^+^ count < 200 cells/mL. It is recommended that HIV-seropositive adults or adolescents (≥ 10 years) with a CD4^+^ count < 200 cells/mL should be screened for CrAg on serum or plasma by reflex laboratory testing (preferred) or clinician-initiated testing. If the clinician-initiated testing is performed, then it is recommended that screening should be restricted to adults or adolescents without prior cryptococcal disease who are initiating or re-initiating ART. For patients with a new positive CrAg result and an LP that rules out CM, oral fluconazole alone as induction therapy should be given (adults 1200 mg daily for 2 weeks). In these patients with a negative CSF CrAg result, ART can be started immediately with fluconazole. Patients diagnosed with CM should be managed as per the latest *Southern African HIV Clinicians’ Society guideline for the prevention, diagnosis and management of cryptococcal disease among HIV-infected persons: 2019 update*.

◦ **Common pitfall: Not performing an LP in all patients who are newly diagnosed as CrAg positive.** The absence of any symptoms of meningitis does not exclude CM; approximately one in three patients with asymptomatic antigenaemia has concurrent CM.

### Isoniazid preventive therapy

#### Key points

➢ Isoniazid preventive therapy should be started at ART initiation or added to the treatment regimen of patients already on ART who have not yet received IPT, once active TB has been excluded.
➢ There is no need to test for latent TB prior to commencing IPT.
➢ Isoniazid preventive therapy should not be started during pregnancy, except in pregnant women where the CD4^+^ count *is* < 350 cells/mL and who are at high risk of death from TB.
➢ Before commencing IPT, active TB infection should always be excluded.

Clinical trials conducted in South Africa and Cote d’Ivoire have shown that IPT has an additive effect with ART in preventing incident TB in HIV-infected patients.^97,98^ In the South African trial, there was a 37% reduction in incident TB when patients receiving ART were prescribed IPT (vs. placebo) for 12 months. This benefit applied irrespective of tuberculin skin test (TST) status, and the trial included patients established on ART. All patients receiving ART should be considered for IPT and screened for active TB using a symptom screen^99^ – IPT should be deferred and investigations should be conducted for active TB if any of the four symptoms (current cough, fever, night sweats or weight loss) is present. In patients receiving IPT, monitoring for neuropathy and hepatitis symptoms should be performed. Routine ALT monitoring is not indicated, but ALT should be tested if hepatitis symptoms occur.

A recent trial of IPT in pregnant women receiving ART, the TB APPRISE study, showed that IPT resulted in worse pregnancy outcomes.^100^ However, this was not confirmed in a larger observational study from the Western Cape, which showed that IPT use was associated with better pregnancy outcomes, and that incident TB was reduced in women on IPT who had CD4^+^ counts < 350 cells/mL.^101^ The duration of IPT is now 12 months irrespective of TST status, as outlined in Table 28.

**TABLE 28:** Indications for isoniazid preventive therapy (provided that there are no tuberculosis symptoms or contra-indications to isoniazid).

Patient category	IPT	Duration
Non-pregnant, regardless of CD4^+^ count	Indicated	12 months
Pregnant women with CD4^+^ count > 350 cells/μL	Not indicated	N/A
Pregnant women with CD4^+^ count < 350 cells/μL (at high risk for TB)	Indicated	12 months

ART, antiretroviral therapy; CD4^+^, cluster of differentiation 4; IPT, isoniazid preventive therapy; N/A, not applicable; TB, tuberculosis.

## 28. Adherence

### Patient readiness for antiretroviral therapy

#### Key points

➢ Each patient commencing ART needs to be prepared for treatment before or during early ART period.
➢ Barriers to adherence (e.g. depression, alcohol use, non-disclosure and food security) and any misconceptions about ART must be identified in the preparation for ART or during the early period of ART.

Preparing patients for lifelong ART with good adherence is a critical component of achieving long-term efficacy and preventing treatment resistance. To accommodate counselling, traditionally two or three visits are required, staggered closely together, before ART. However, it is now considered acceptable to do some of the counselling during early ART rather than delaying initiation (same-day initiation is described in section 6). Prolonged delays in commencing ART should be avoided. Antiretroviral therapy should be delayed only if concerns about adherence are severe enough to outweigh the risk of HIV disease progression.

The patient should be provided with details regarding:

- the benefits of ART
- that ART is a life-long therapy
- the importance of good adherence
- a list of ART side effects relevant to the drugs they will use, including what to do and who to contact if serious side effects occur
- viral load monitoring on ART.

The counselling approach should also ensure that the patient has a good understanding of HIV (the virus, the potential clinical complications and transmission) and should cover safer sex practices and address issues related to reproductive health (i.e. family planning, contraception, condom use and pregnancy). Clinicians should check family-planning choices at follow-up visits and ensure adequate access to safe and effective contraception. It is important to discuss the concept of ‘Undetectable = Untransmittable’ with patients and ensure that they have a correct understanding of this concept and that ART will only prevent onward transmission if there is optimal adherence with VL suppression.

Active depression, other mental health issues or substance abuse should be detected actively and treated. A personal treatment plan should be formulated for each patient, specifying drug storage, strategies for missed doses and how to integrate taking medication into their daily routine. The patient must be made aware of scheduling in terms of clinical follow-up.

Disclosure of HIV status (to a partner and/or other household members) should strongly be encouraged; it is an important determinant of treatment adherence and assists in the provision of patient-directed support. Disclosure also identifies exposed contacts for screening and support. This issue needs to be handled carefully in situations where disclosure may have harmful consequences, particularly for women. The patient should be encouraged to join a support group and/or identify a treatment ‘buddy’. However, neither disclosure nor support group participation is a prerequisite for good adherence and should not be a reason for deferring ART. Clinicians should ensure that they have the contact details of each patient and their treatment buddy.

◦ **Common pitfalls:**
  ◦ Delaying ART because the patient has not completed three clinic visits or not disclosed his or her HIV status.
  ◦ **Not outlining the goals of ART with the patient**. These are to:
    ■ provide maximal and durable suppression of VL
    ■ restore and preserve immune function
    ■ reduce HIV-related infectious and non-infectious morbidity
    ■ prolong life expectancy and improve quality of life
    ■ prevent onward transmission of HIV
    ■ minimise adverse effects of the treatment.

### Support and counselling

#### Key points

➢ Success of ART hinges on how well the tablets are taken; at least 90%, preferably more, of treatment doses need to be taken.
➢ Support should be provided to ensure high levels of treatment adherence.
➢ None of the commonly used first- and second-line options have meaningful food restrictions.
➢ Delayed dosing is rarely a problem; even if out by many hours, most of the drugs have long half-lives, and patients should be encouraged and supported to take their dose once they remember to do so in these instances.
➢ Disclosure is not a prerequisite for ART.
➢ Heavy alcohol use may affect adherence and may potentiate ART hepatotoxicity and other hepatic pathology; however, responsible alcohol use is not prohibited in patients established on or starting ART.

Patients should be informed about the benefits of ART and that side effects are usually minor and transient, or manageable. They should be given a treatment plan, specifying the drugs to be used (with names and details including the appearance of each drug, when and how they are to be taken, and a brief indication of anticipated side effects and toxicity).

The causes of poor adherence are often complex and linked to social issues. Common causes are outlined in Table 29.

◦ **Common pitfalls:**
  ◦ **Not informing patients about the benefits of ART.** This includes not only reduced mortality and morbidity, but also prevention of HIV transmission.
  ◦ Not informing patients that side effects are usually minor and transient, or manageable.
  ◦ Not advising patients on how to deal with delayed dosing.
  ◦ **Not providing patients with a treatment plan specifying the drugs to be used.**

**TABLE 29:** Possible reasons for poor adherence.

Individual	Provider	Medication
Depression	Stock-outs	High pill burden
Alcohol or substance use	Inaccessible clinics (both in place and time)	Frequent dosing (more than once per day)
Non-disclosure	Poor communication (not patient-centred)	Adverse effects
Inadequate treatment literacy	-	-
Adolescence	-	-
Following pregnancy	-	-
Food security	-	-
Work-related issues (shift work)	-	-
Social problems (stigma and poor social support networks)	-	-
